# Supplementary material for: Robust mapping of spatiotemporal trajectories and cell–cell interactions in healthy and diseased tissues
Source: Nat Commun. 2023 Nov 25;14:7739. doi: 10.1038/s41467-023-43120-6 (PMC10676408; doi:10.1038/s41467-023-43120-6)
Supplement: Supplementary file 1 — Supplementary Information [file 41467_2023_43120_MOESM1_ESM.pdf]

Supplementary Figures

Authors: Duy Pham, Xiao Tan, Brad Balderson, Jun Xu, Laura F. Grice, Sohye Yoon, Emily F. Willis, Minh Tran, Pui Yeng Lam, Arti Raghubar, Priyakshi Kalita de Croft, Sunil Lakhani, Jana Vukovic, Marc J. Ruitenberg, Quan H. Nguyen

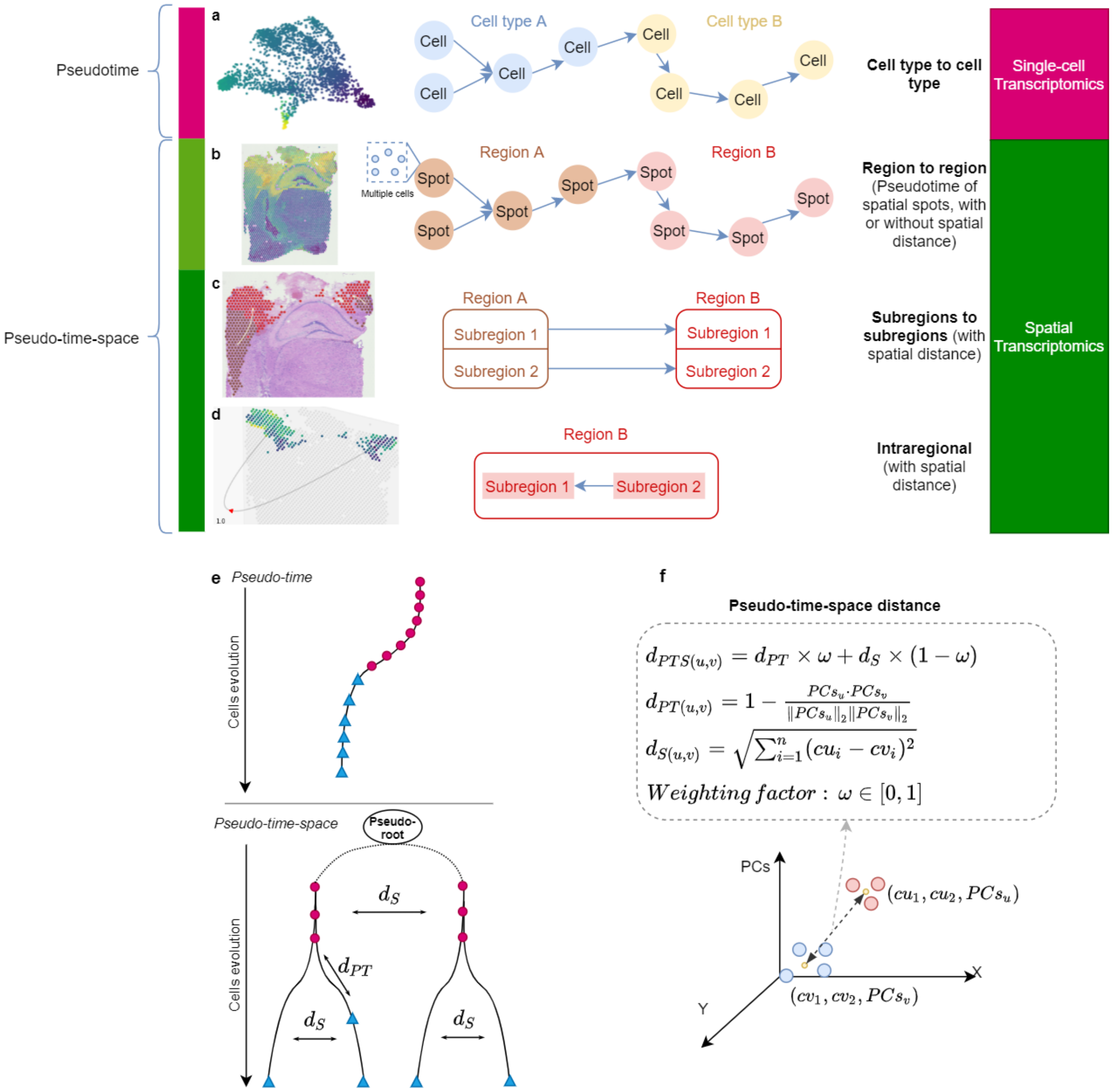

Figure 1. **Pseudo-time-space (PSTS) concept and algorithm for spatial trajectory inference**. **a**, Pseudotime is a popular concept in single-cell RNA sequencing data analysis, where it is used to construct trajectories for studying transition processes from one cell type and/or state into another. **b**, PSTS adds space context and spatial variation to model pseudotime-space (PSTS), and was specifically developed for spatial omics data. PSTS can be applied to study dynamic processes across (sub-)regions (space) across a given (set of) tissue sections. **c,d**, Spatial trajectories can be modelled between adjacent (**c**) or separated (intraregional; **d**) sub-regions (sub-clusters). **e**, Pseudotime and PSTS in the context of lineage tracing. The addition of spatial information allows for more detailed trajectory mapping. **f**, Basic PSTS formulae are visualised in three dimensions, where the *x* and *y* axes represent spatial data and the *z* axis pseudotime.

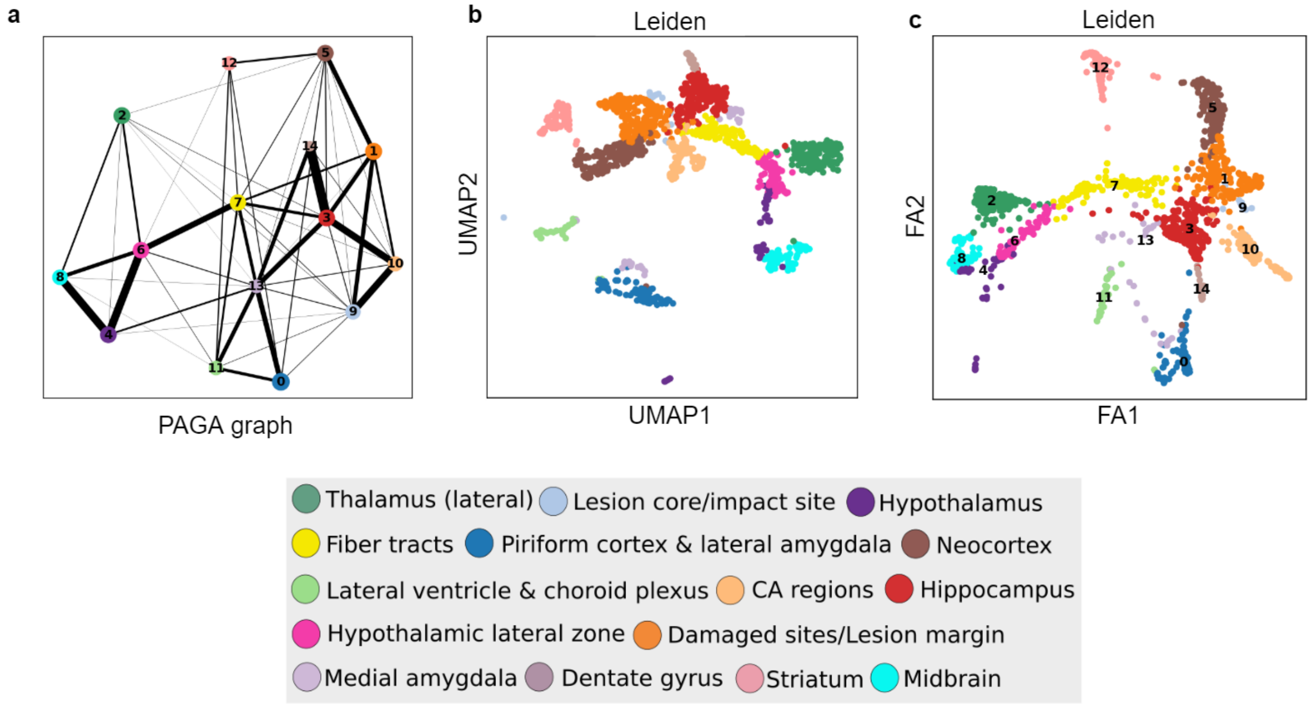

Figure 2. Cluster visualisation and connectedness for the Visium mouse TBI dataset. Identified ST clusters are represented either in graph form or in a reduced dimensional space; connections between nodes are intermediate outputs from stLearn, before graph optimisation. **a**, PAGA graph of mouse TBI sample (3 days post-injury). Without spatial optimisation, the PAGA graph shows that most nodes are connected, highlighting the need for a more specialised analysis method to model trajectories in ST data. **b-c**, Visualisation of clustering results in UMAP (b) or Force Atlas (FA) space (c), suggesting distinct clusters represent different anatomical regions, but do not necessarily show direct relationships between cell types and/or states across the tissue section (refer to Fig. 2c for locations of the clusters across the tissue).

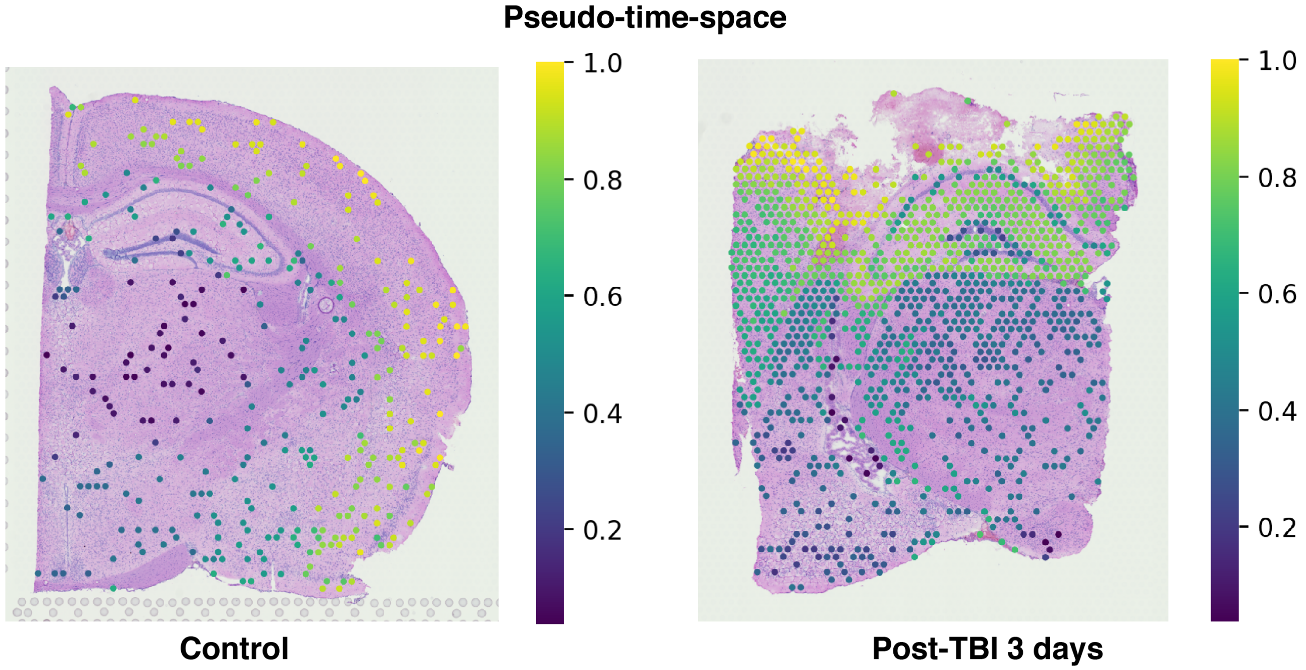

Figure 3. Comparison of pseudo-time-space (PSTS) values for naïve (uninjured) mouse brain and mouse TBI samples. PSTS values are mapped to the spatial context (microglia-containing spots). Note that the TBI sample shows a strong change and gradient from the deeper (ventral) side of the brain toward the dorsally-located impact site.

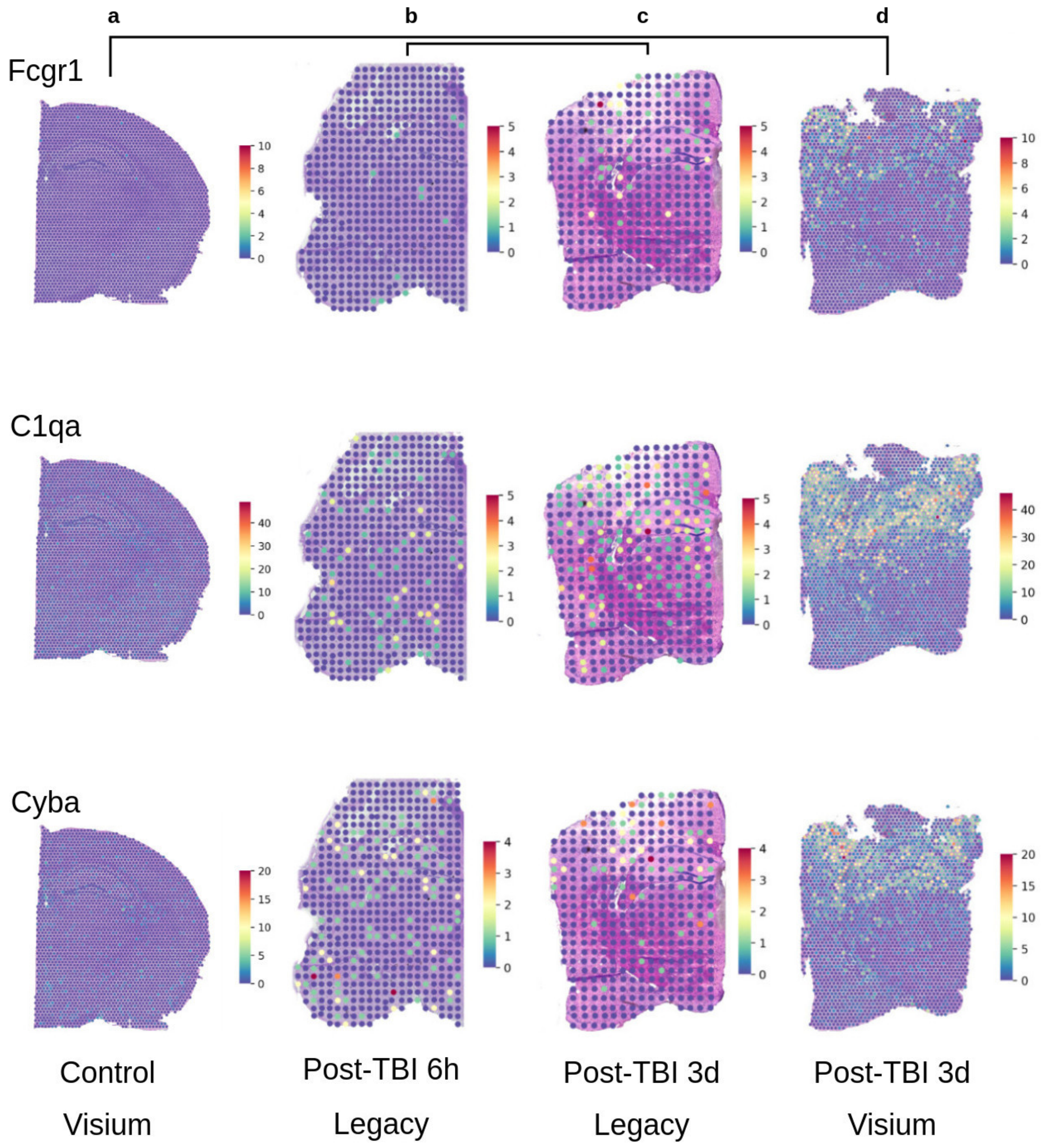

Figure 4. Visualisation of genes associated with microglial activation across time and space. The expression patterns of $Fcgr1$,$C1qa$, *and Cyba* are shown for two different platforms (Legacy ST and Visium) and time points (6 hours and 3 days post-TBI). **a**, Coronal brain section of an uninjured control mouse using the Visium platform. **b**, Legacy ST data for a TBI sample at 6 hours post-injury. **c**, Legacy ST data for a TBI sample at 3 days post-injury (the impact site is at the middle top part of the tissue). **d**, Visium ST data for a TBI sample at 3 days post-injury (tissue is from the same block shown in **c**). Note that the scales are matching for samples from the same platform (compare **a** *vs.* **d**, and **b** *vs.* **c**, top bars).

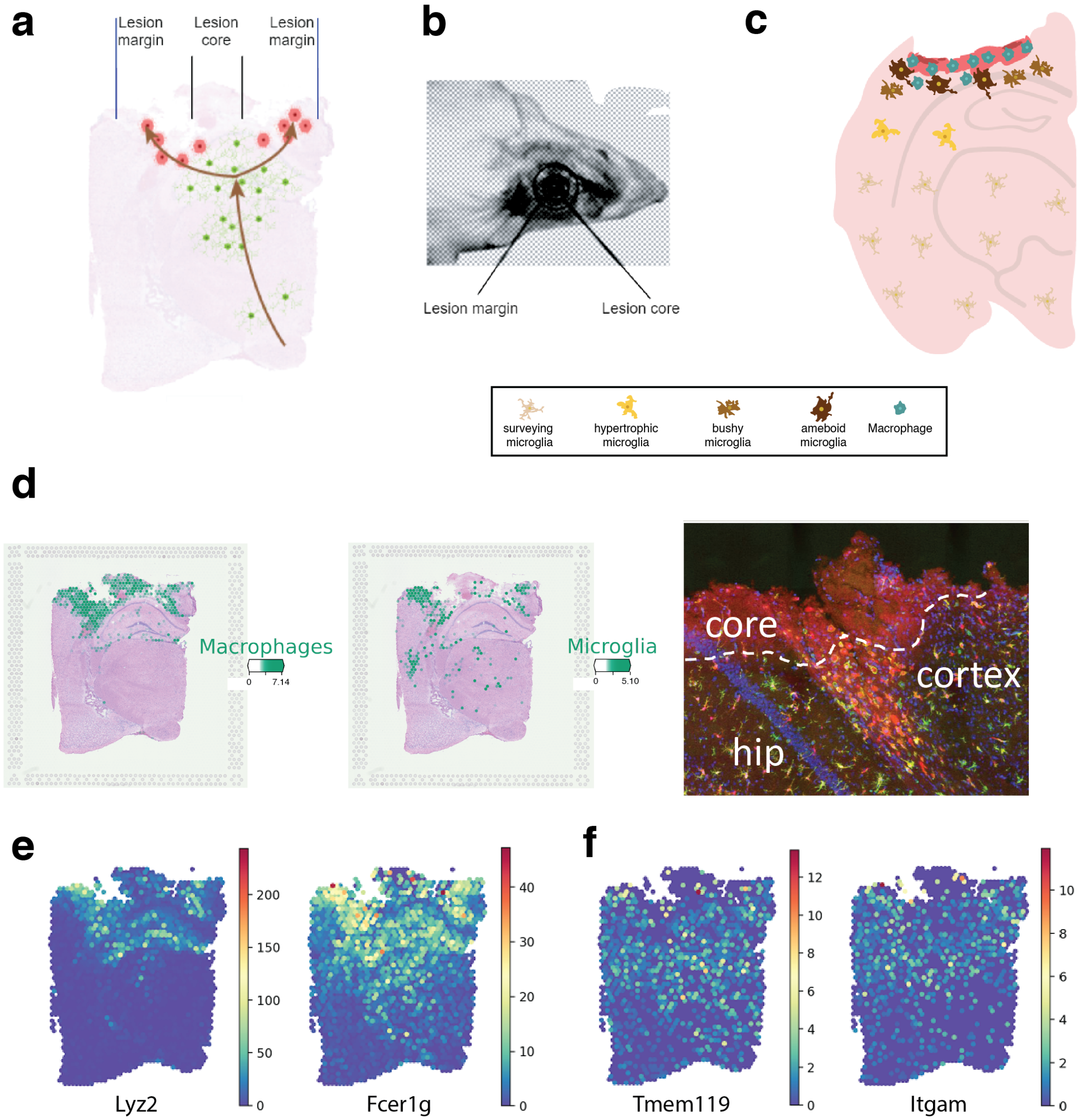

Figure 5. Evaluation of microglial responses to traumatic brain injury (TBI). **a-c**, Spatial branching response concept. **a**, Diagram depicting that the microglial response to TBI is more pronounced at the lesion margin (refer to Fig. 2f), and also that these cells are mostly missing from the lesion core (see **d**). **b**, Projection visualisation of regions defined as lesion margin and/or core in the controlled cortical impact (TBI) model. **c**, Expected distribution pattern of microglia activation in TBI and associated morphological change (from surveying (homeostatic) to hypertrophic, bushy and/or amoeboid). **d**, Cell type deconvolution result for macrophages (left) and microglia (middle) based on ST data from TBI mice (3 days post-injury). Note that microglia are mostly absent from the lesion core. This was also independently validated in histological studies (right, confocal photomicrograph) where genetically marked tdTomato-positive (red) microglia, co-stained with Iba-1 (green), can be seen accumulating along the lesion margins whilst they are mostly lacking from the lesion core; cell nuclei are show in blue; hip, hippocampus. **e**, Expression of *Lyz2* and *Fcer1g* as more generic marker genes for microglia/macrophages. **f**, Expression of *Tmem119* and *Itgam* as marker genes for microglia, suggesting again a microglia distribution that is mostly at the lesion margins, not the lesion core.

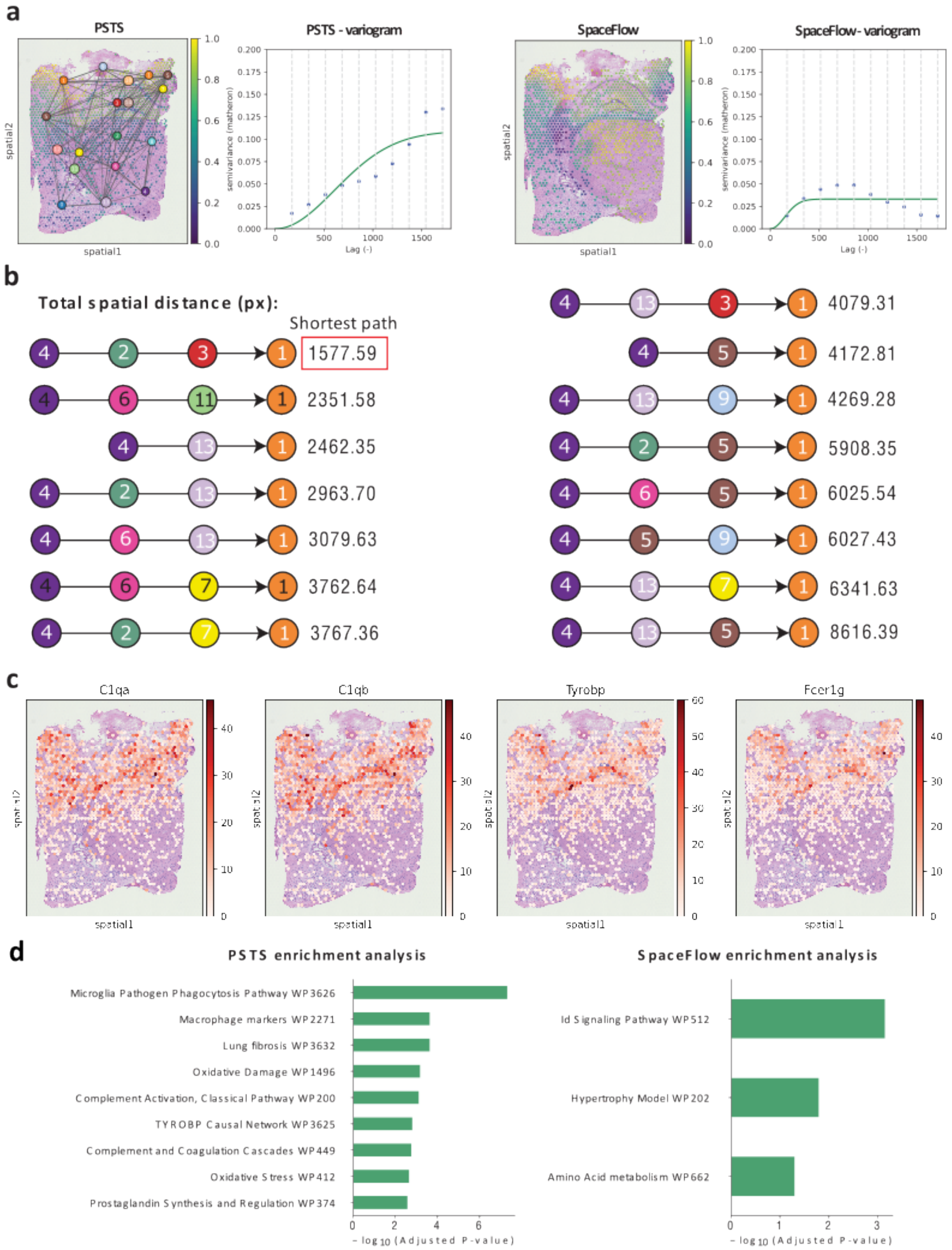

Figure 6. Comparison of stLearn PSTS and SpaceFlow methods around spatial pseudotime scores and associated downstream analyses. a. For PSTS, a spatial-PAGA graph was constructed (*left*), and the distribution of its pseudotimespace values examined using a variogram across all spots; the semivariance values of PSTS ranged from 0 to 0.135. The distribution of pseudotime values from SpaceFlow and the associated variogram is shown on the right; SpaceFlow's semivariance values ranged from 0 to 0.05. **b.** Top-15 shortest paths from cluster 4 to cluster 1 (i.e., from lowest to highest PSTS value), as determined by the minimum spanning tree algorithm. The weighting score for each path was calculated based on the total spatial distance between the nodes. Among these paths, the highlighted result of 1578 pixels (px) for $4 \to 2 \to 3 \to 1$ represents the route with the shortest physical spatial distance between cluster 4 and cluster 1. **c.** Expression of microglia-related transition genes (from PSTS analysis) C1qa, C1qb, Tyrobp, and Fcer1g within the spatial context. **d.** Enrichment analyses results, conducted using the genes identified as significantly correlated with PSTS (*left*) or SpaceFlow (*right*) pseudotime scores. Only PSTS enrichment analysis revealed the presence of microglia-related pathways within the mouse TBI ST-seq dataset.

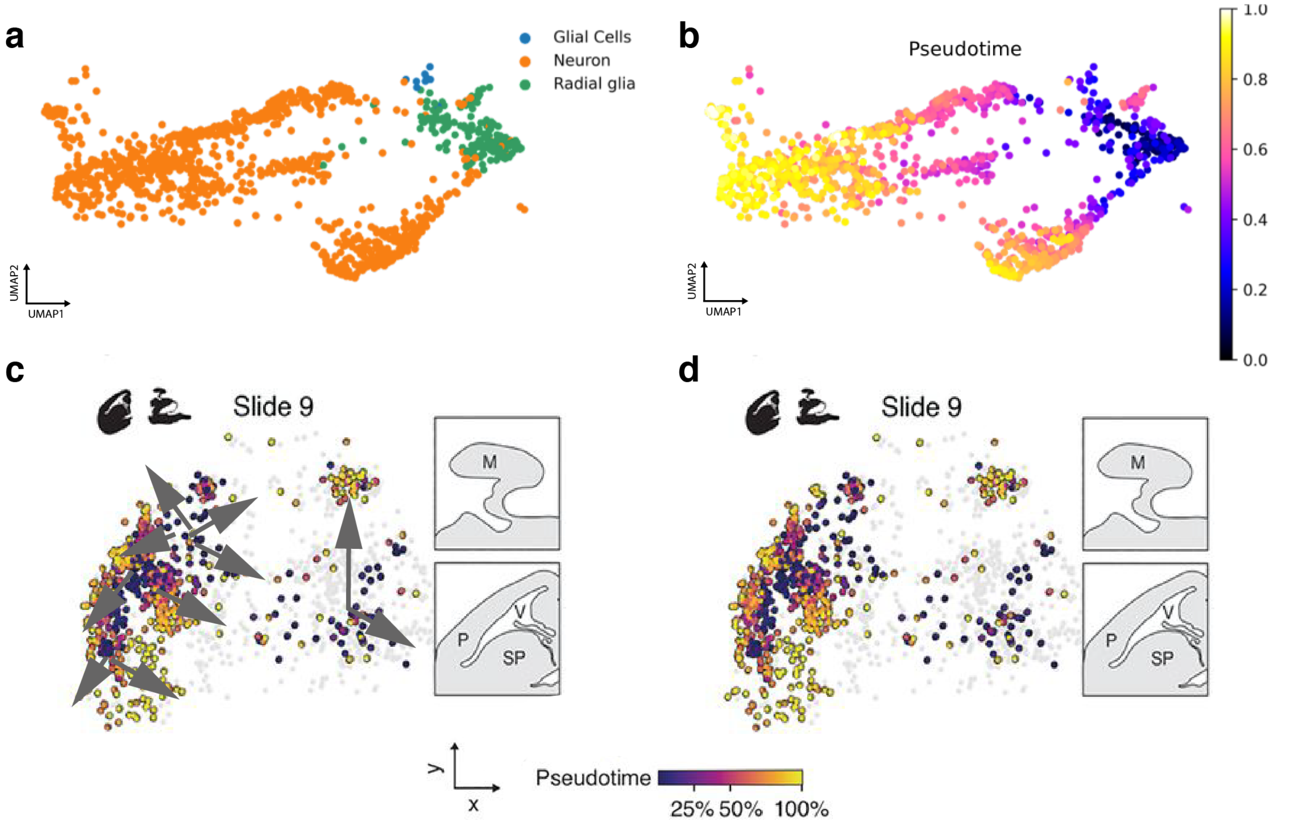

Figure 7. stLearn PSTS can add spatial trajectory information to the analysis of embryonic mouse brain development. **a**, Annotated cells in UMAP space. Three cell types are shown: glial cells (blue), neurons (orange) and radial glia (green). **b**, Pseudotime values in UMAP space, reconstructed based on the diffusion pseudo-time method (part of PSTS) to assess the reproducibility of the result in sci-Space study, which showed transcriptional changes at the early developmental states by radial glia and glial cells compared to differentiated neurons, but no spatial trajectory could be drawn. **c, d**, Prediction of the spatial trajectories, within tissue context, for the Slide 9 sample that added a layer of trajectory prediction with transitioning directions in the neuronal development in the sci-Space study. Slide 9 results are shown with (**c**) and without (**d**) the spatial trajectories; pseudotime values were adopted from the original study and we added the predicted spatial trajectory by PSTS. (M, midbrain; P, pallium; SP, subpallium; V, ventricle)

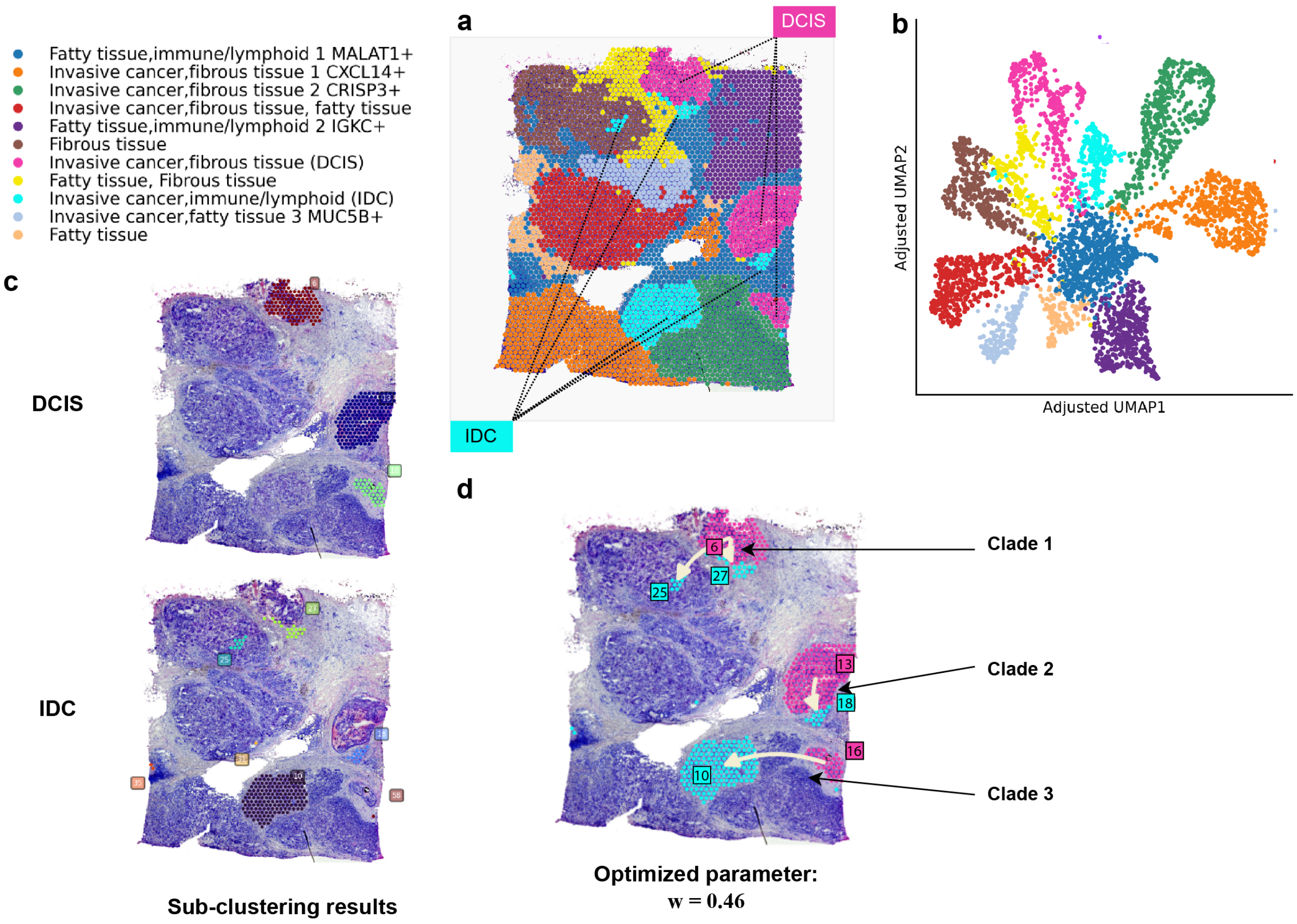

Figure 8. **stLearn PSTS connects DCIS-IDC sub-clusters in the Visium human breast cancer dataset**. **a**, Clustering results (*right*) with corresponding annotations of cell types for each cluster (*left*). **b**, Visualisation of clustering results in UMAP space, with coordinates adjusted for spatial distance so that two transcriptionally similar spots that are also nearer in tissue space become more similar. **c**, Sub-clustering results of ductal carcinoma *in situ* (DCIS; top) and invasive ductal carcinoma (IDC; bottom). **d**, stLearn PSTS predicts three cancer clades, with the arrows showing the transition between non-invasive (pink) and invasive (blue) ductal carcinoma regions.

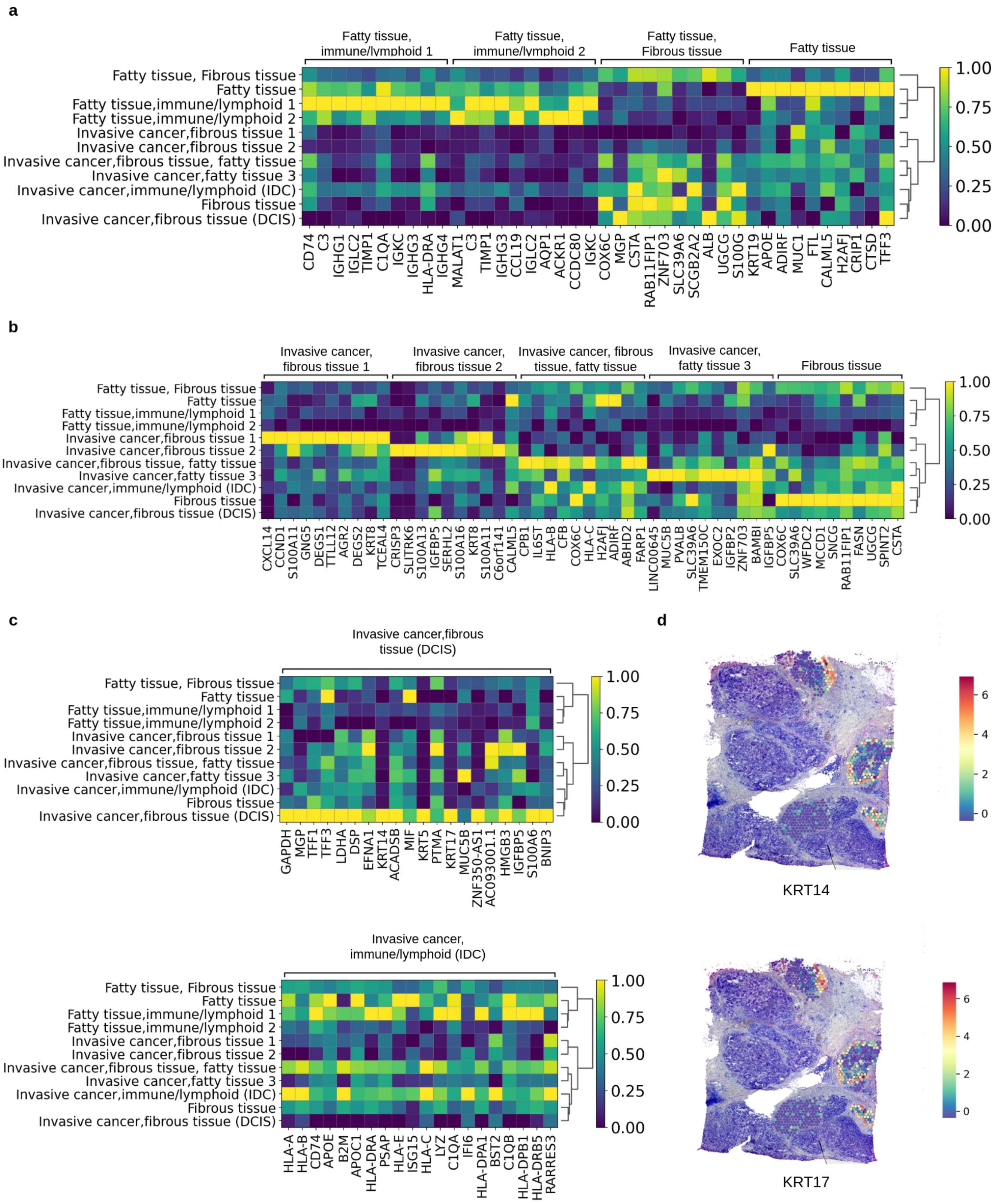

Figure 9. Top gene markers associated with breast cancer clusters. Cluster annotations used for trajectory analysis are displayed. **a-c**, Heatmap plots showing scaled gene expression values for the top 10 differentially expressed marker genes for each of the eleven clusters found by stLearn (cluster names refer to clusters shown in Fig. S8a; genes for the 11 clusters are split across 4 heatmaps for readability). **d**, Visualisation of *KRT*14 and *KRT*17, two DCIS markers whose expression is localised to the edges of the DCIS clusters.

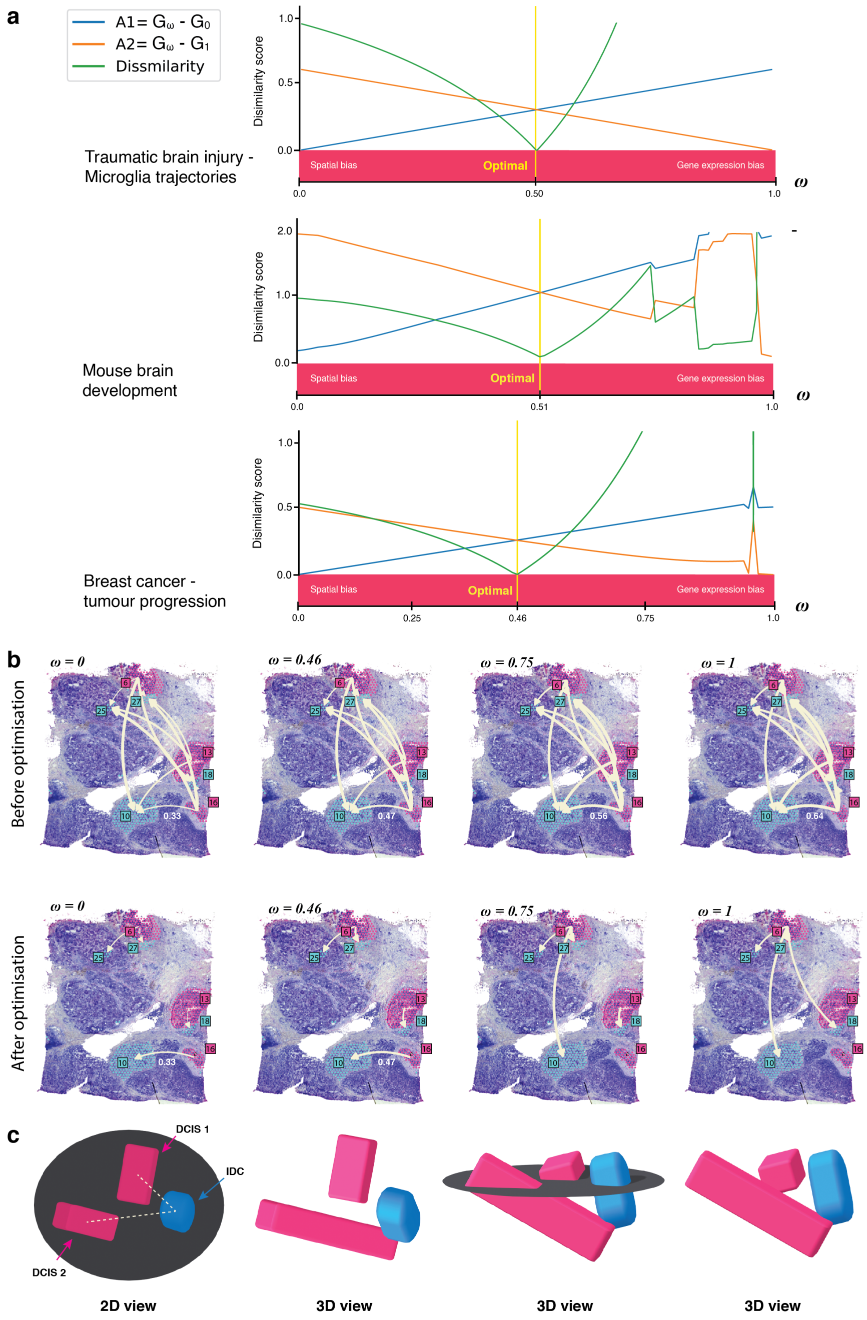

Figure 10. Optimisation of ω parameter for spatial trajectory analysis. **a**, Grid-search procedure for determining the optimal weighting for ω, as implemented for three spatial datasets, namely experimental TBI, mouse brain development and human breast cancer progression. Spectral distances between G_0 and G_W (A1; blue line), and between G_1 and G_W (A2; orange line) are plotted across all possible values of ω between 0 (spatial information only) and 1 (gene expression information only) in 0.01 increments. A1 shows the difference of the resulting trajectory compared to when only gene expression information is used, and A2 the difference compared to using spatial information only. The dissimilarity score ($|1 - A1/A2|$) is plotted in green. The ω value with the minimum dissimilarity score, that is where A1 is most similar to A2 (vertical yellow line), is then selected as the optimal value for this parameter, that is, where a balance between spatial and gene expression information is reached. This optimal ω value is then used to construct a graph connecting spatial nodes, which is further optimized via a minimum spanning tree algorithm to form a trajectory (see PSTS Methods section). **b**, Case study on the Visium breast cancer dataset showing spatial trajectories resulting from different ω values, before and after optimisation. Spatial trajectories reconstructed with $\omega = 0.46$ provide the optimal integrated balance between spatial and gene expression information in both the weighted edges and final structure of the graph. **c**, Simulation of a 3D scenario involving two DCIS (purple) and one IDC (blue) clusters. Perspective bias is evident in the 2D view, with the distance from DCIS 1 to the IDC being closer than from DCIS 2. As easily appreciated from all 3D views, however, the correct adjacency is to DCIS 2 not DCIS 1, which highlights the importance of gene expression information for establishing connectedness.

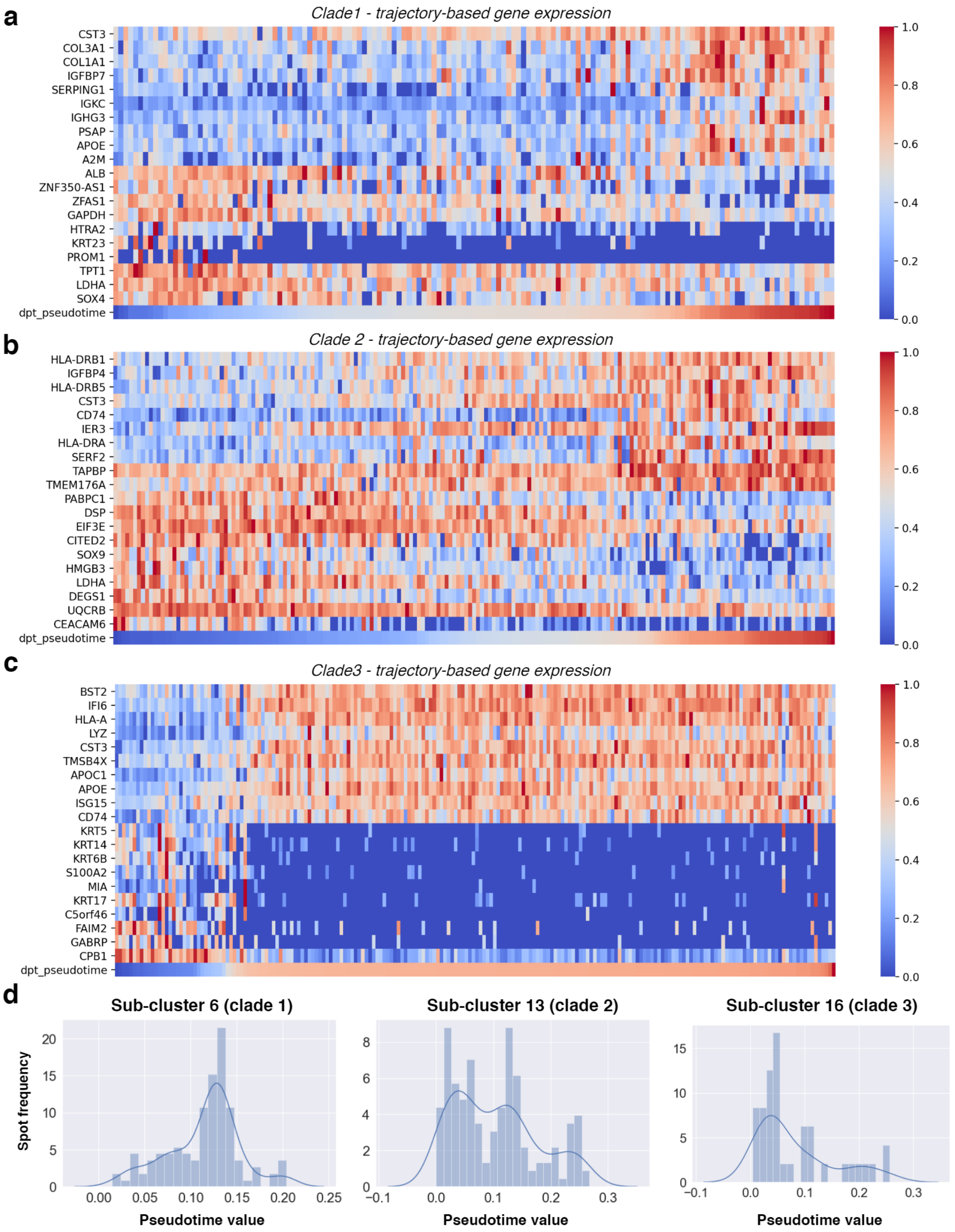

Figure 11. **Transition markers of the breast cancer DCIS-to-IDC spatial trajectories**. Top 10 up- and downregulated transition markers of (**a**) clade 1, (**b**) clade 2, and (**c**) clade 3, (the clades shown in Fig. 3e,f and S8d). Markers were predicted by calculating Spearman's correlations between gene expression and PSTS values. Upregulated genes are positively correlated with PSTS values (i.e. increasing along the trajectory; Spearman's coefficient > 0.3, p-value < 0.05), and downregulated genes are negatively correlated with PSTS values (i.e. decreasing along the trajectory; Spearman coefficient < -0.3, p-value < 0.05). **d**, Differential distribution of pseudotime values in each DCIS sub-clusters (i.e. sub-cluster 6, sub-cluster 13 and sub-cluster 16), suggesting the three clades are biologically defined.

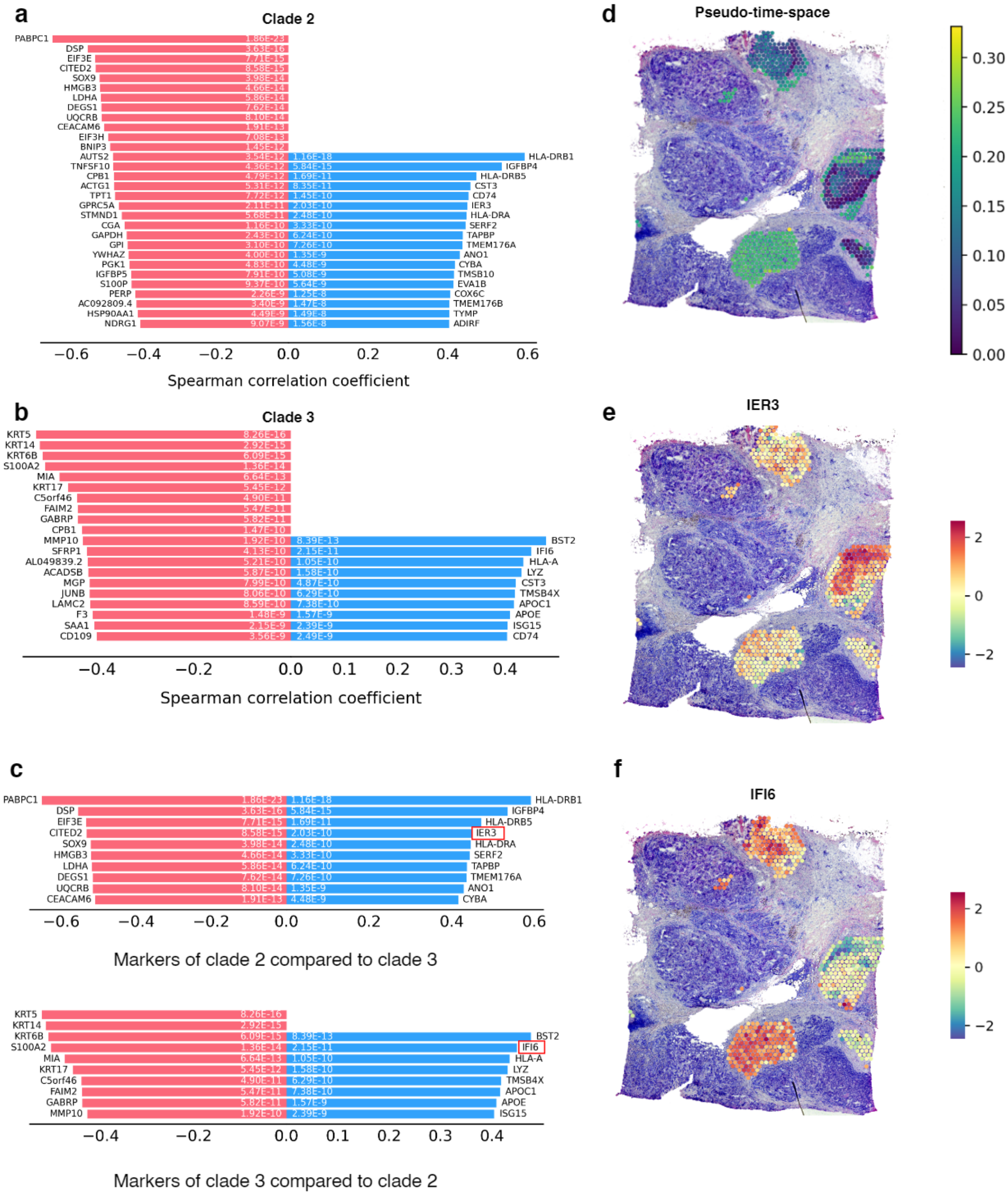

Figure 12. Transition markers of spatial trajectories for breast cancer clades 2 and 3. Top 30 down- (left, red) and upregulated (right, blue) transition markers of clade 2 (**a**), and clade 3 (**b**). Genes listed are those identified and shown in Fig. S11. **c** Comparison of up- and downregulated marker genes between clades 2 and 3. The top 100 up- and downregulated markers associated with each clade were compared to extract only those genes that are unique to a given clade. Plots show Spearman's correlation coefficients (x-axes) and p-values (within bars) for specific gene markers that differentiate clade 2 (top) and clade 3 (bottom). Red boxes highlight example genes shown in **e** and **f**. **d-f**, Visualisation of PSTS values within their tissue context for DCIS and IDC clusters, and normalised gene expression plots of selected marker genes associated with clade 2 (*IER3*; **e**) and clade 3 (*IFI6*; **f**). Note the consistent patterns of PSPT values in clades 2 and 3 moving from low to high (see **d**), and that the spatial expression patterns of positively correlated marker genes for clade 2 and clade 3 also move from low to high expression, as shown in **e** and **f**.

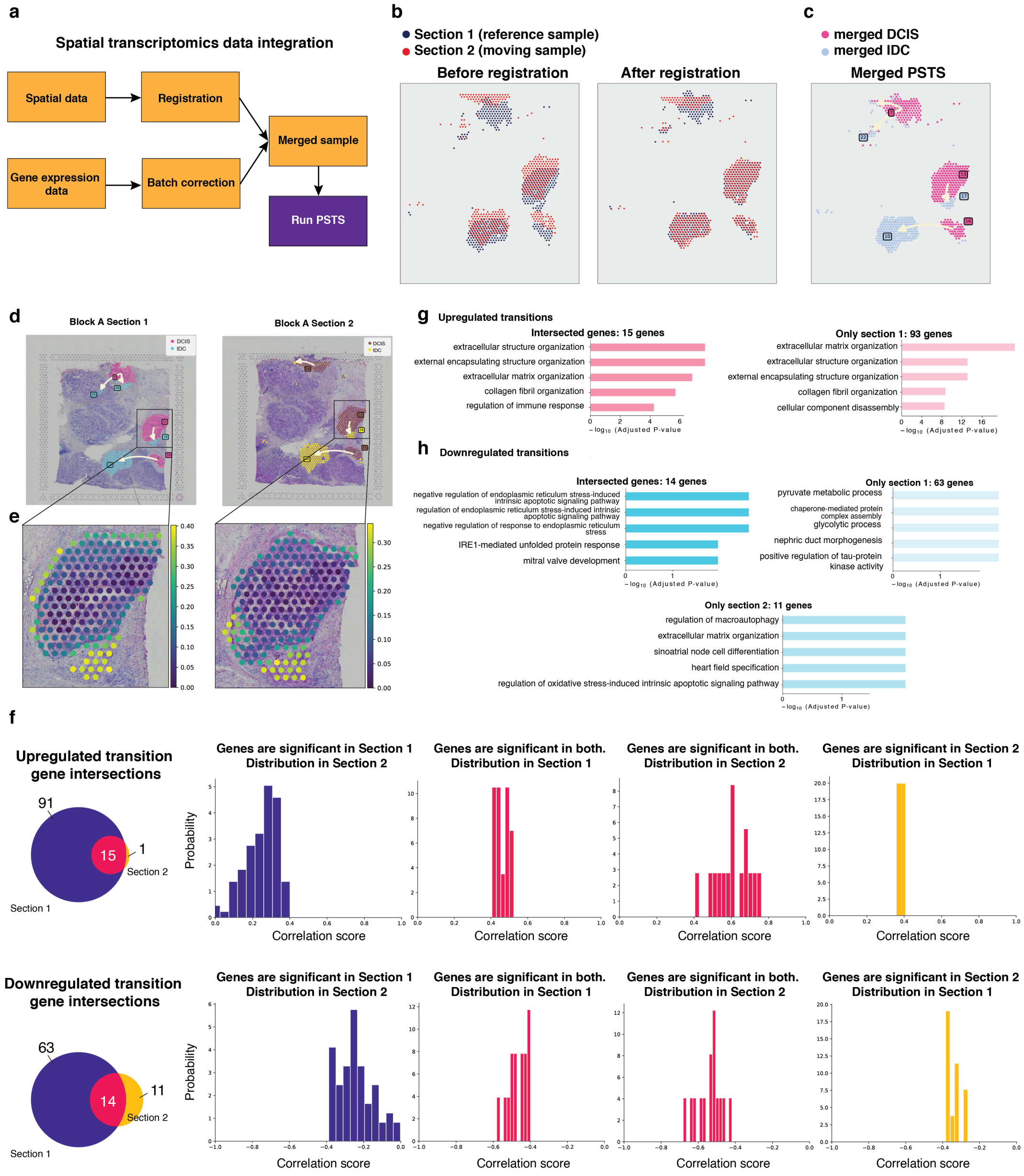

Figure 13. **Spatial trajectory inference using multiple datasets**. **a-c** Registration-based strategy for inferring common trajectories between sections; **d-h** trajectory inference through a shared driver gene strategy. Adjacency sections 1 and 2 of the Visium human breast cancer dataset (sample block A) are used. **a**, Workflow for image registration and data integration. **b**, Image registration of two spatial datasets. **c**, PSTS results for the merged dataset in the common coordinate system. **d**, Spatial trajectories resulting from applying the PSTS method to sections 1 and 2 individually. **e**, Cropped example view of DCIS to IDC cancer progression. Note that the spatial pattern of pseudo-time-space values has the same trend in each section, that is, from the inside of the duct to the edge, and then to the IDC part. **f**, Venn diagrams show unique and shared transition genes for the trajectories in **d**. Histograms show correlation scores for up- (*top row*) and downregulated (*bottom row*) driver genes that were either shared (red) or not shared (blue and yellow) as part of the common trajectory. Note that genes not shared between sections still mostly followed the expected pattern of up- (positive correlation) and downregulation (negative correlation), but they did not pass the correlation threshold of 0.4 or -0.4, respectively. Also note that the shared driver genes consistently show stronger correlation scores. **g**, Enrichment analysis for upregulated transition genes in two cases: intersected genes between both block A sections (*left*), and transition genes identified from section 1 only (*right*). Note that identified pathways are consistent across, with extracellular matrix-related biological processes dominating. **h**, Enrichment analysis for downregulated transition genes in three cases: intersected genes between both block A sections (*top left*), transition genes identified only in section 1 (*top right*), or those in section 2 only (*bottom*). In general, the significance values were lower for downregulated genes (less DEGs per pathway), meaning more weakly matched pathways in each case. Pathways associated with apoptotic signaling were nonetheless found in all three cases (7th GO term for 'section 1 only' genes; data not shown), thus also providing evidence here for consistency.

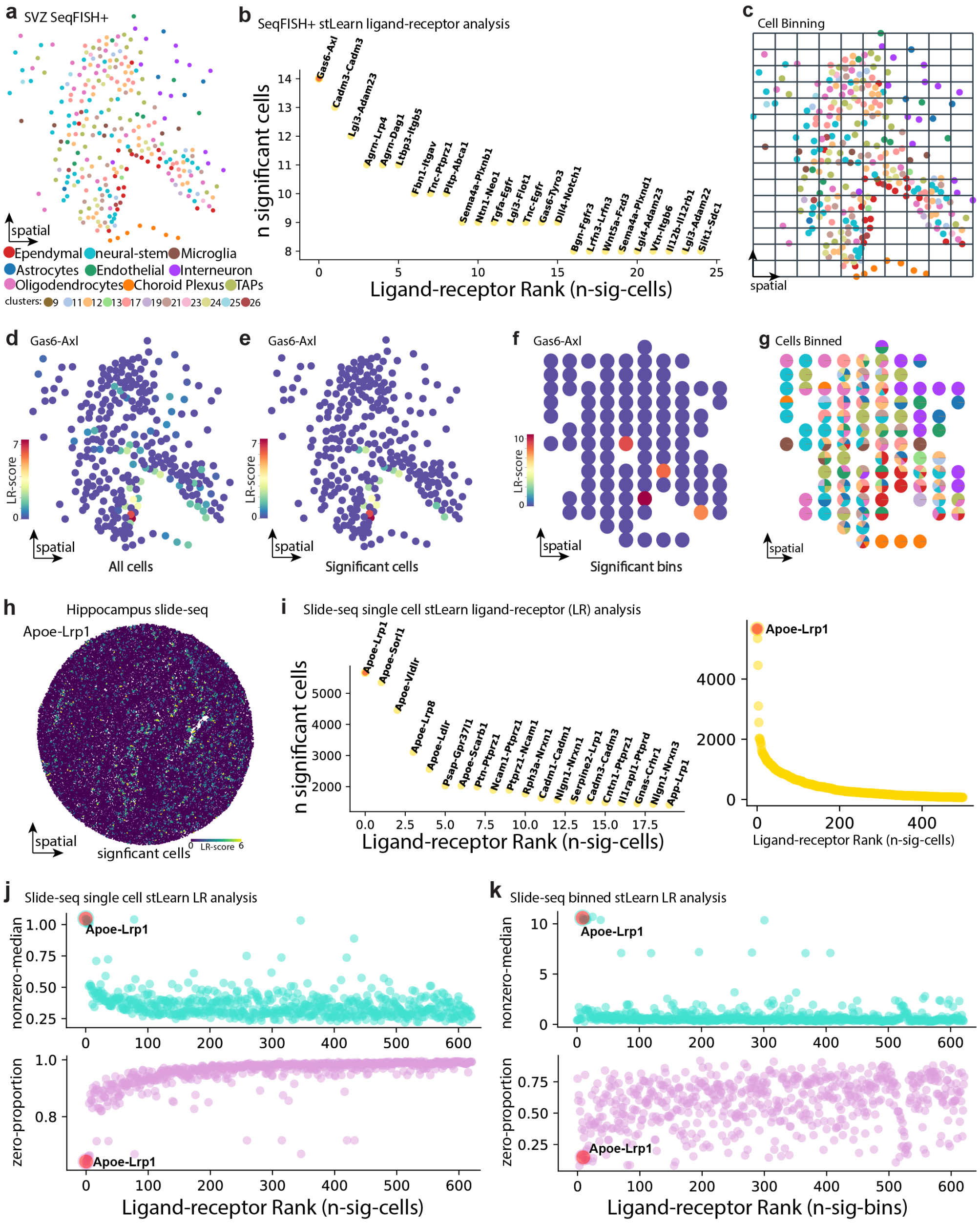

Figure 14. stLearn SCTP analysis scales to millions of cells. **a**, Sub-ventricular zone (SVZ) seqFISH+ single-cell spatial transcriptomics data. Colours show cell types or cluster numbers. **b**, Scatter plot showing the top 25 LR pairs from the seqFISH+ data, as identified by stLearn SCTP, with *Gas6-Axl* being the top-ranked pair. **c**, SeqFISH+ data with overlaid bins, an approach successfully used to improve computation speed with comparable results (see **f, g, k**). **d, e**, Spatial distribution of all (**d**) and significant (**e**) stLearn LR scores for *Gas6-Axl*. **f**, Significant scores for *Gas6-Axl* on binned data. **g**, SeqFISH+ data binned according to **c**; pie plots indicate binned cell types. **h**, Hippocampus Slide-seq data showing significant *Apoe-Lrp1* scores. **i**, Top LRs for the Slide-seq data, showing that *Apoe-Lrp1* is top-ranked pair (left). **j** Slide-seq analysis scatter plots for all LR pairs, illustrating relationship between LR rank and expression level (top), and the proportion of 'zero expressing' cells (bottom). **k**, Same as **j**, except that analysis was conducted on binned data. A similar spatial significance between single-cell and binned data is observed, demonstrating method scalability.

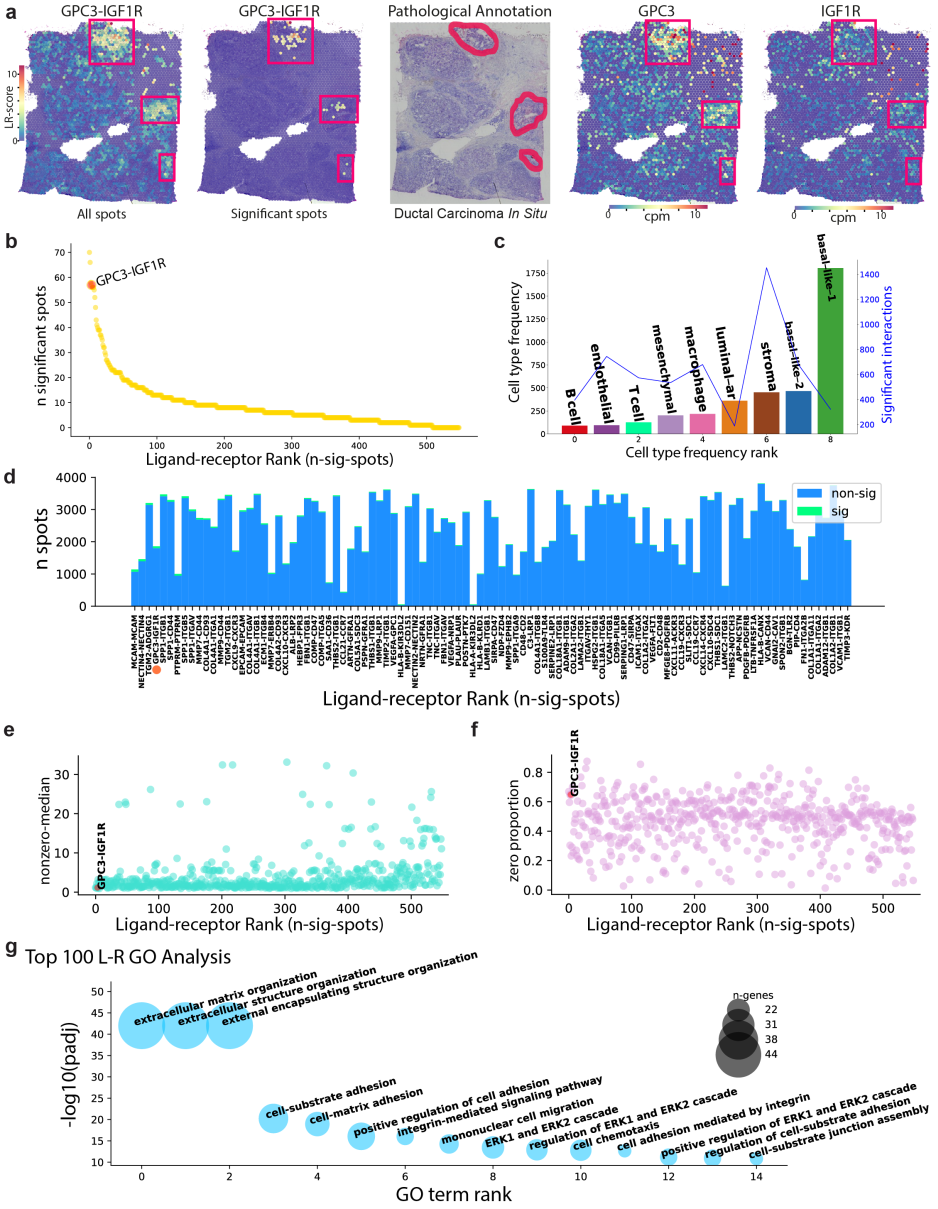

Figure 15. stLearn ligand-receptor interaction analysis effectively accounts for technical biases. **a**, (left to right) Gpc3-Igf1r stLearn ligand-receptor (LR) scores across the Visium human breast cancer tissue; LR scores subsetted to significant spots; H&E image of the breast cancer tissue section marked up based on pathologist annotations of Ductal Carcinoma *In Situ* (DCIS) regions, highlighted in red; the counts per million (cpm) normalised expression for *GPC3* and *IGF1R* per spot. **b**, Scatter plot of all LRs ranked by the number of significant spots; *GPC3-IGF1R* is one of the top interacting pairs (and the highest when only considering the Invasive Ductal Carcinoma region). **c**, Bar chart and line plot on the same *x*-axis; bars show the frequency of each cell type in the Visium breast cancer dataset, while the line indicates the number of significant cell-cell interactions by a given LR pair (*GPC3-IGF1R* in this case). No correlation between cell type abundance and the number of interacting spots is observed. **d**, Bar graph showing the top 50 most highly interacting LR pairs from stLearn SCTP analysis. Pairs are ranked on the *x*-axis from the greatest number of significant spots to the lowest, with *GPC3-IGF1R* marked up (orange dot). The total number of spots with an LR pair score is shown on the *y*-axis, with green showing significant spots and blue showing non-significant spots. Note that the number of significant spots is not correlated with the total number of spots with LR scores. **e**, Scatter plot showing the nonzero-median expression of LR pairs across all spots on the *y*-axis and the ranking of LR pairs by the number of significant spots on the *x*-axis (high rank means low number of significant spots). No correlation of LR expression level and LR significance is observed. **f**, Scatter plot showing equivalent *x*-axis as in **e**, but the *y*-axis now indicates the proportion of spots with zero gene expression for the LR pair. No correlation is observed between expression frequency and significant LR rank, indicating the method robustly controls for the frequency of LR expression across the spots. **g**, The top 15 (ranked by -log10(adjusted p-value)) significant Gene Ontology terms that are enriched based on the top 100 LR pairs. Several cell-cell signalling terms are observed, including the *ERK1/2* cascade, which is a key signalling pathway in breast cancer progression.

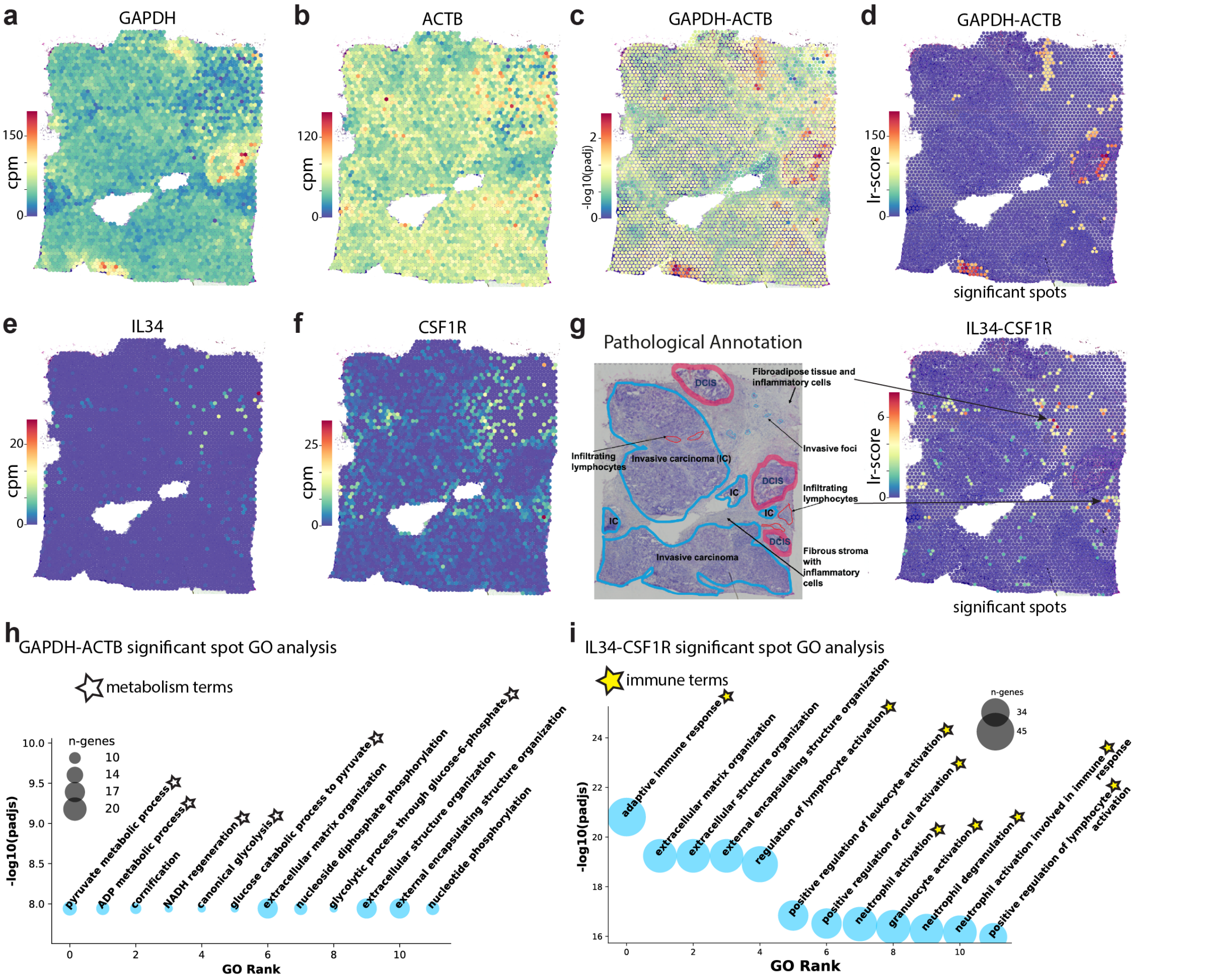

Figure 16. Downstream interaction analysis to assess significant LR interactions. **a-d**, stLearn ligand-receptor (LR) analysis for a non-interacting gene pair (*GAPDH-ACTB*), which exemplifies a low, non-significant false positive rate for this pair (3.4%). Gene expression (in counts per million reads, cpm) is shown for *GAPDH* (**a**) and *ACTB* (**b**) across the tissue. **c**, -log10 (adjusted p-values) from the permutation test for *GAPDH-ACTB* across each spot throughout the entire tissue. **d**, Spots with significant *GAPDH-ACTB* LR scores (false positive detection - 3.4%) among all spots. **e-g** Analysis of *IL34-CSF1R* interactions (immune-related LR pair). Gene expression values are shown in cpm for *IL34* (**e**) and for *CSF1R* (**f**) across all spots. **g**, Pathological annotation (left) relative to the locations of spots with significant *IL34-CSF1R* interactions (right), showing that significant spots predominantly occur in areas marked up as having immune cell infiltration. **h**, Downstream GO pathway analysis for genes differentially expressed in spots with significant *GAPDH-ACTB* LR scores. Stars indicate pathways related to cell metabolism. **i**, Similar to **h**, but now showing results from GO pathway analysis for regulated genes in spots with significant *IL34-CSF1R* LR scores. Stars indicate significantly enriched pathways relating to immune responses.

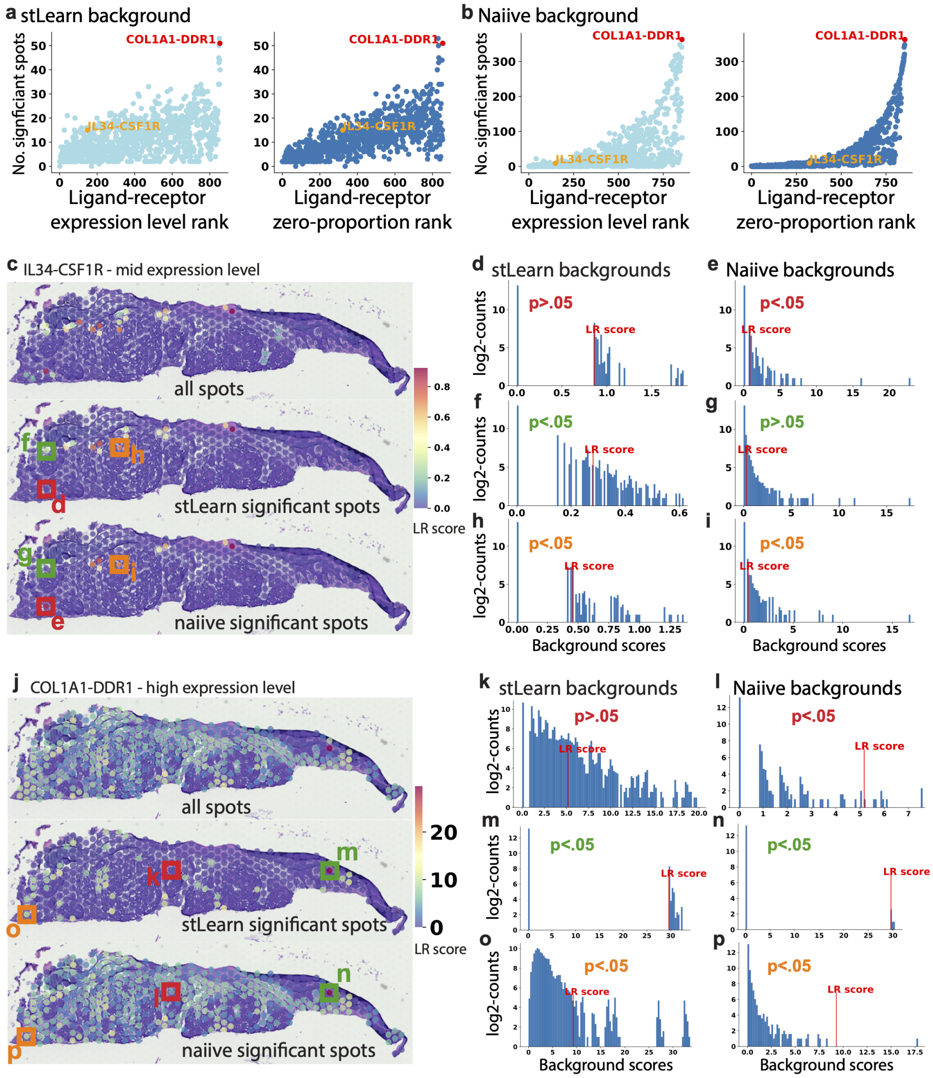

Figure 17. Effect of background selection on stLearn SCTP ligand-receptor interaction predictions. **a**, stLearn SCTP, which accounts for background gene-gene pairs with the same expression range as the query ligand-receptor (LR) pairs, as applied to Visium human skin cancer data. (*left*) Scatter plot with the number of significant hotspots for LR pairs on the *y*-axis and LR expression level rank on the *x*-axis. Examples of high- (*COL1A1-DDR1*) and mid- (*IL34-CSF1R*) expressing LR pairs are shown in red and orange, respectively. A rank of 1 means the lowest LR expression level. (*right*) Same as left, but with the *x*-axis now displaying the LR ranking by the proportion of zeros. **b**, Same as **a**, except that significant LR hotspots have now been called based on a naïve background approach, which does not consider the expression levels of random background gene pairs compared to the query LR pair. A strong correlation between very abundant LR expression level (those ranked above 700th) and the number of significant spots is observed with the naïve background; the stLearn SCTP approach (shown in **a**) markedly reduces this effect. **c**, LR scores for the example LR pair with mid-level expression, *IL34-CSF1R*, showing all spots with LR scores (top), stLearn SCTP significant spots (middle), and significant spots using the naïve background (bottom). Coloured squares labelled with letters indicate areas where the same spots with the background generated by either stLearn SCTP or the naïve approach are shown for comparison, highlighting the difference between significant versus non-significant spots using the two methods. **d-i**, Histograms showing the distribution of background LR scores for the mid-level expressing *IL34-CSF1R* pair for either stLearn SCTP (*left column*)or naïve approach (*right column*) for the individual spots highlighted in **c** (boxed areas); Exact p-values are shown on each respective histogram. A one-sided permutation test was applied to compute the probability of the selected LR having a higher score than the overall distribution of random background scores, using stLearn SCTP (see methods). In this case, p-values are calculated for two exemplar LR pairs (IL34-CSF1R and COL1A1-DDR1). For each LR pair, different approaches for generating the background LR-score distributions, so the correction for multiple testing is not required. Red vertical lines show the *IL34-CSF1R* LR scores for particular spots. **j**, Equivalent to **c**, but now highlighting the highly expressing LR pair *COL1A1-DDR1*. **k-p**, Equivalent to **d-i**, except that the histograms now link to spots highlighted in **j**, and with backgrounds generated for *COL1A1-DDR1*. Overall, the naïve background leads to almost all spots being considered as significant for highly-expressed LR pairs, while the stLearn background can be seen to adjust for the LR expression level to call significant regions of co-expression and/or interaction.

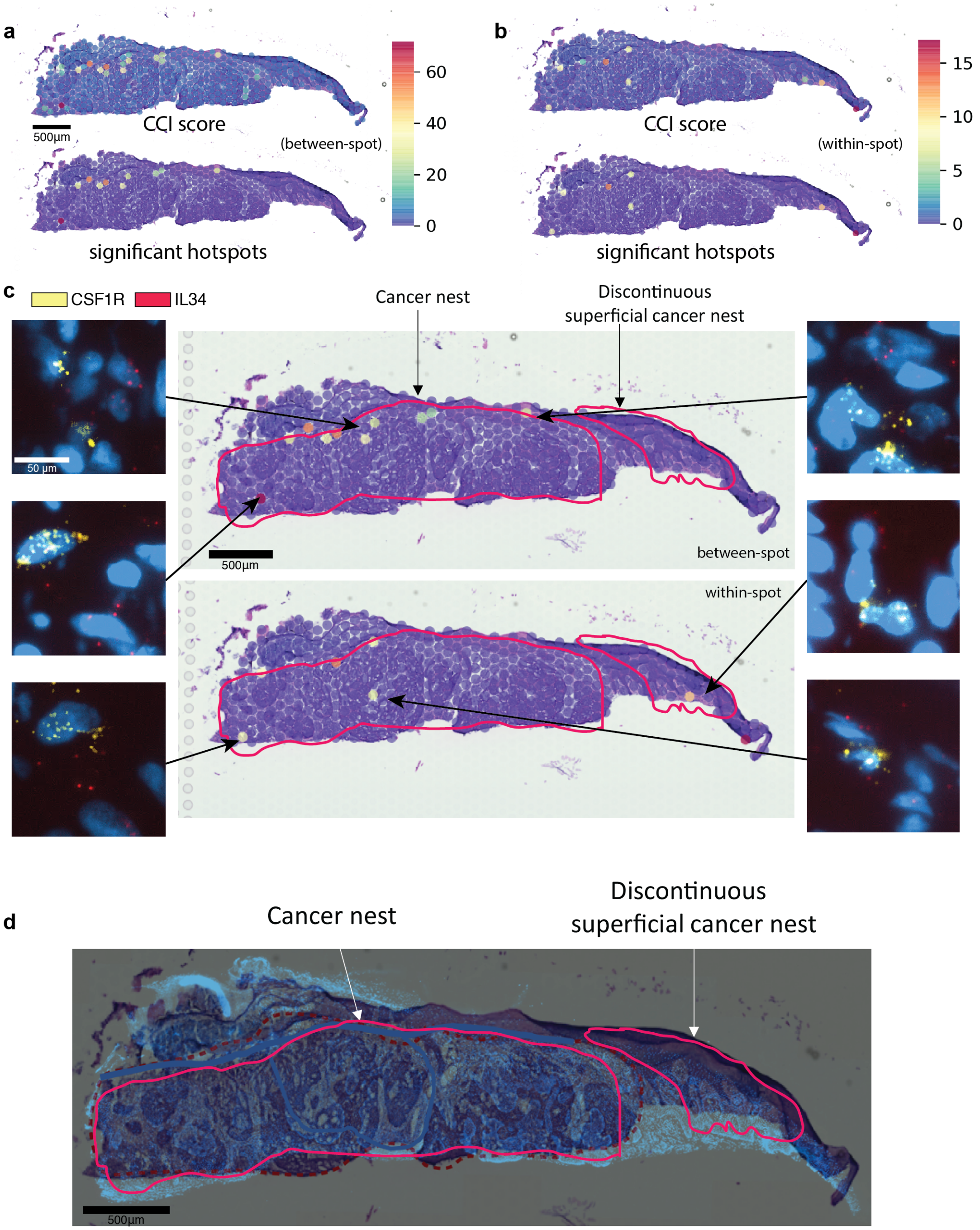

Figure 18. RNAscope validation of stLearn SCTP ligand-receptor analysis. **a**, cell-cell interaction (CCI) scores for the *IL34-CSF1R* LR pair across the human skin cancer tissue (*top*), and significant spots post statistical testing (*bottom*) using between-spot mode. **b**, Same as **a** but now using stLearn SCTP in within-spot mode. **c**, Significant CCI spots from stLearn SCTP analyses (centre panels) compared to RNAscope data (1,179 spots from one tissue section) for corresponding spots in an adjacent tissue section. Confocal microscopy images at single-cell resolution show the highly sensitive and accurate detection of *IL34* ligand (red dots) and *CSF1R* receptor (yellow dots) by RNAscope. Red outlines shows pathological annotation, based on H&E image. Note that *IL34-CSF1R* CCI events mostly occur along the border between the cancer nest and normal tissue regions. **d**, Image registration of RNAscope tissue section (DAPI-stained image) and the H&E image from the Visium ST section. Notably, an exact overlap is not possible because the two adjacent sections are not completely identical.

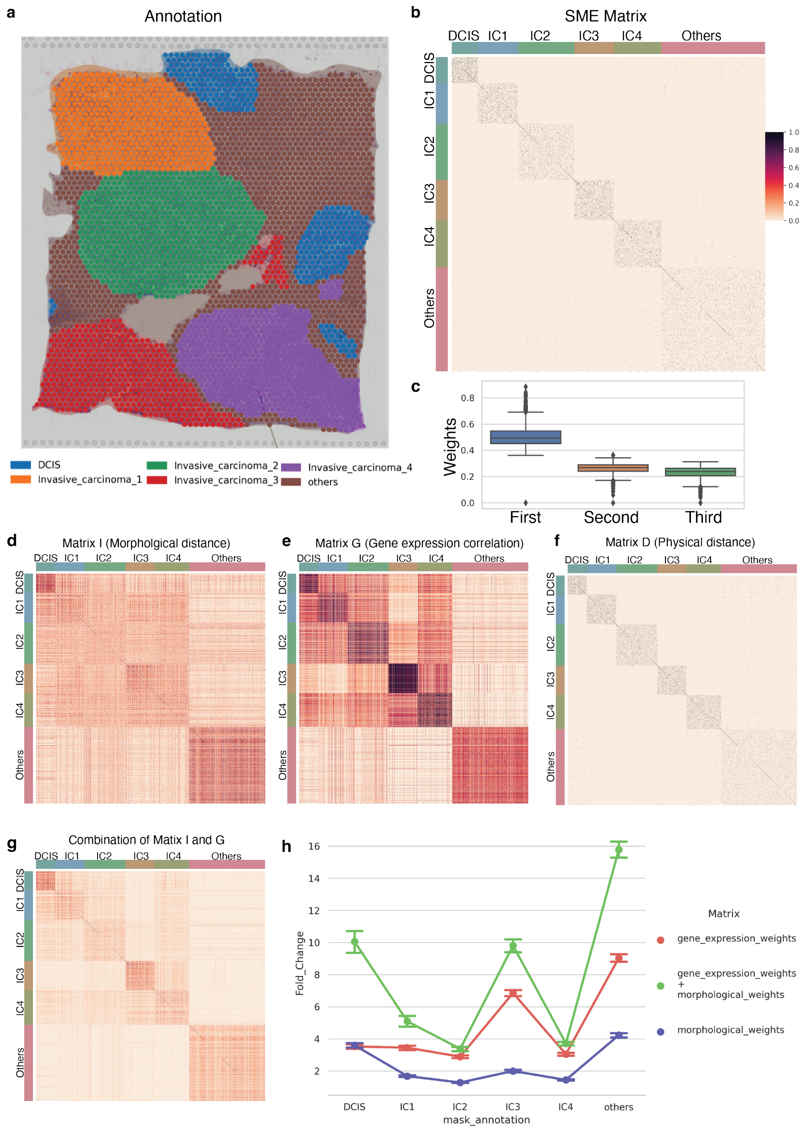

Figure 19. Additive value of integrating ST data types for clustering. **a**, Six general categories of cell types and/or regions were identified in a human breast cancer dataset, labelled based on pathological annotation. **b**, Heatmap of the integrated stSME weights combining (I), (G), and (D). The stSME matrix is used for selecting three spots that are most similar to a given query spot, to then subsequently being selected as the reference spots (refer to Methods). Most weights in the stSME matrix will be near 0, but the top three spots are used as in **c**. Pairwise weights calculated for each of the spatial spots across all spots are shown. For all rows and columns, spots are grouped into categories as in **a**. The colour gradient shows the weight ranging from 0 to 1. **c**, The distribution of three weights for the reference spots used for imputation (all spots (n=3,813) from the breast cancer tissue section). Here, three reference spots were always selected as the spots that were most similar in terms of morphology, spatial location, and gene expression. The sum of the weights of three spatial spots is 1. **d-g**, Similar to **b**, but showing the weights for each component of the three data types, that is, morphological cosine similarity (I) in **d**, gene expression Pearson correlation (G) in **e**, and spatial (physical) distance (D) in **f**; the combined imaging and gene expression matrix is shown in **g**(see also Methods section). **h**, The relative fold-changes of weights of spots within each cluster compared to all other remaining spots (e.g. mean of DCIS spots divided by the mean of all other spots), (all spots from the breast cancer tissue section (n=3,813); data with error bars show mean values +/- SEM). The fold-change indicates the level of the weights contributing to the differences between one cluster and all others. The higher the fold-change, the more discriminative power the weights contribute to the identity of the cluster. The general increase when combining gene expression and morphology weights demonstrates the added power for distinguishing biologically relevant clusters within the dataset. DCIS = Ductal carcinoma in situ; IC = Invasive carcinoma.

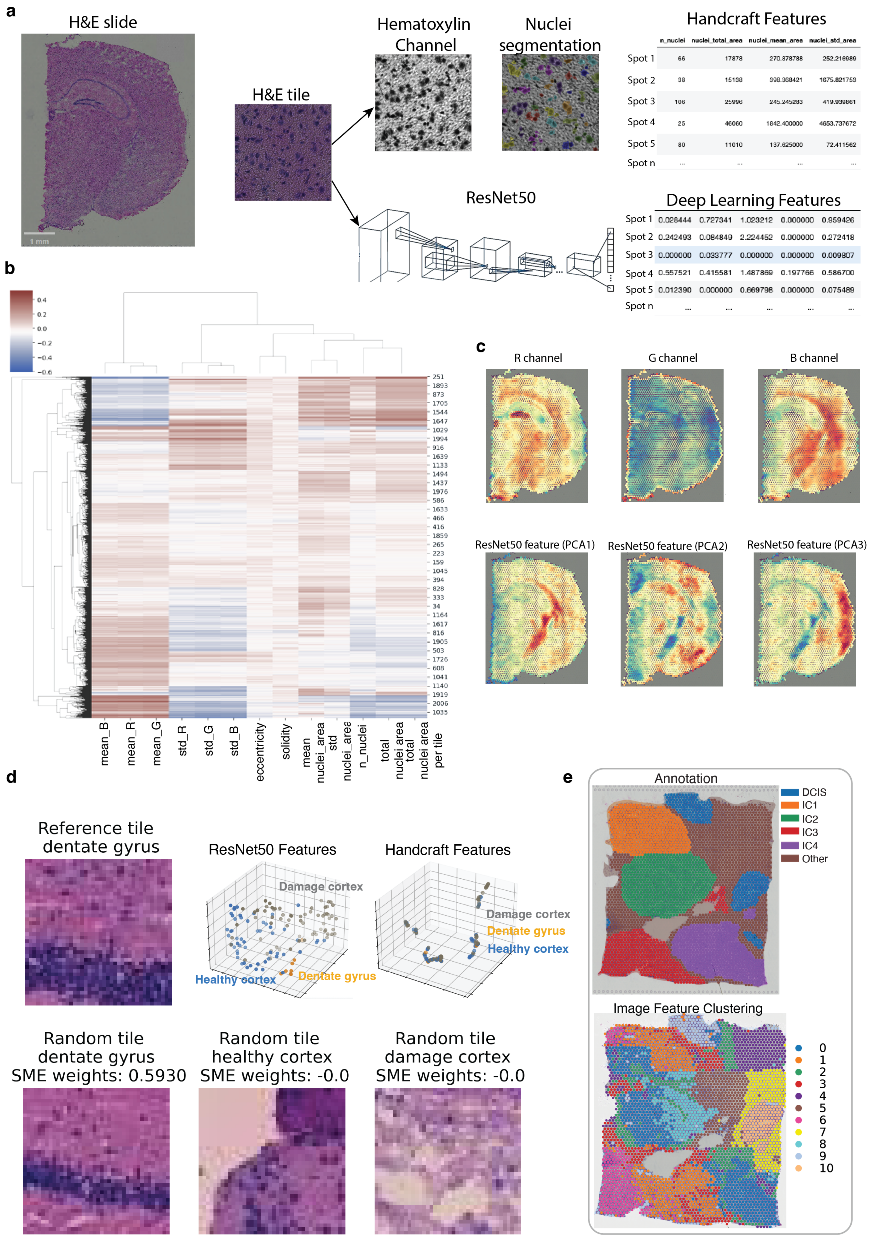

Figure 20. Value and interpretability of image features used in stLearn. **a**, Two approaches were used to extract image features, enabling the interpretability of information contained within a H&E image per spot and/or across the tissue section. Handcraft features (as listed in **b**), An example of such downstream analysis is the association between the number of cells, or the size of nuclei, and the gene expression measured in each spot ($n_{slide} = 1$, $n_{tiles} = n_{spot} = 2,702$). Deep learning features (2048 for each tile) are those extracted from the ResNet50 model. **c**, Comparing information from just the colour channels (R, G, B) alone (*top row*) suggests that the higher representation features of the image (i.e., ResNet50 features, projected to PCA space; *bottom row*) encode information with a higher contrast between regions than in the raw image input. **d**, Model interpretability analysis, focusing on the morphological similarity parameter. The mouse TBI dataset was used here for the assessment of morphological similarity between two spots (two image tiles). Cosine similarity values between feature vector outputs from the neural network for randomly selected spots (tiles) within the same anatomical category (dentate gyrus), or between different categories (healthy or damaged cortex), are shown; stSME weights are cosine values. Three-dimensional scatter plots show the high level representation (feature vectors) of the imaging tiles from the neural network (top row, *middle*), or those from the handcraft features (top row, *right*). Each dot is one image tile, and the colours are labelled by and/or representative of different anatomical regions. Note that ResNet50 but not handcraft image features could distinguish and/or separate out the dentate gyrus, and also lesioned from spared (healthy) cortex, on the reduced 3D tSNE space. **e**, ResNet50 features could also capture key functional regions in the human breast cancer tissue, suggesting again that these features contain functional information (compare to clustering analysis shown in Fig. 6g and Fig. S21c).

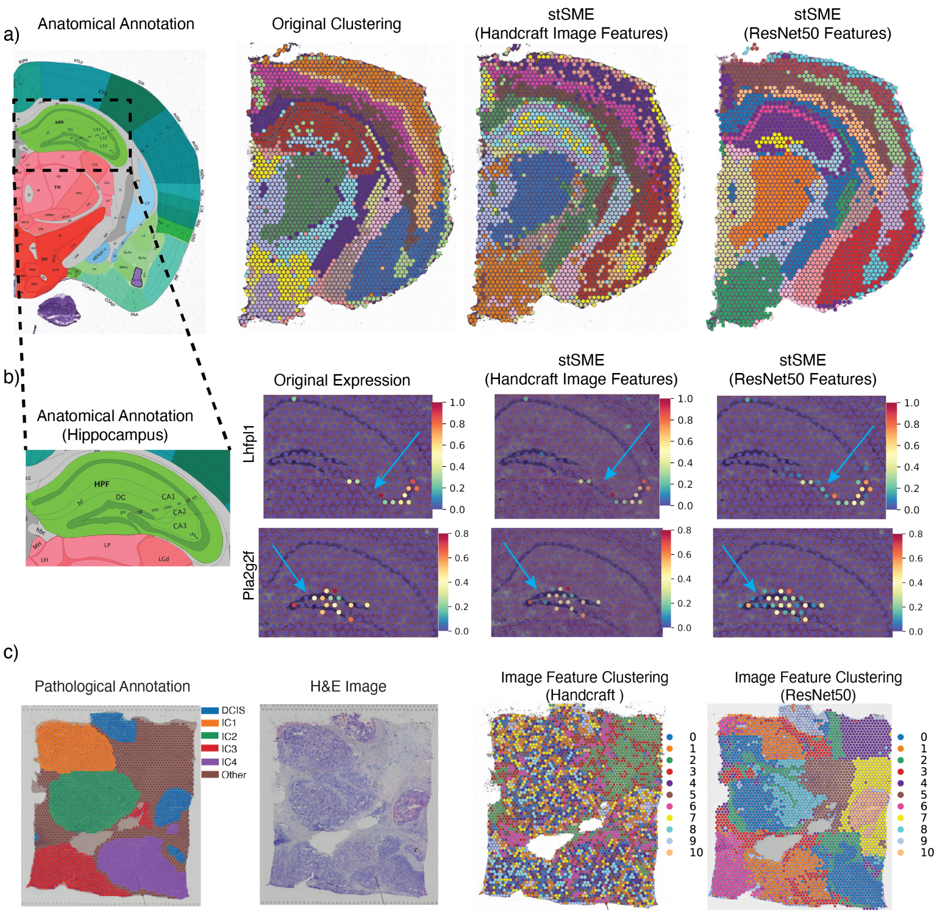

Figure 21. Capturing of ResNet50 image features enables superior performance of stSME. a Clustering results for the Visium mouse brain dataset, comparing the anatomical annotation (first column) with the original spatial data (second column, baseline without morphological information), stSME but with use of handcraft features to capture basic morphological information (third column), and stSME using ResNet50 features (fourth column). Note that clustering results are less noisy when ResNet50 features are used (as compared to handcraft image features), and also that only stSME with ResNet50 features was able to accurately identify smaller anatomical structures and/or sub-regions such as the dentate gyrus (DG), cornu ammonis (CA) 1 and CA3 regions. **b** Imputation results for *Lhfpl1* (*top row*) and *Pla2g2f* (*bottomw row*), marker genes for the CA3 region and dentate gyrus, respectively; the anatomical reference is again shown on the left. Note that only stSME with ResNet50 (*right*) but not handcraft features (*middle*) successfully imputed missing values (blue arrows) compared to baseline (original expression, *left*). **c** Comparison of clustering performance (based on image features only), as applied to the Visium human breast cancer dataset; left two panels show the pathological annotation and original H&E image, respectively. Right two panels show Louvain clustering using either handcraft image or ResNet50 features as the input.

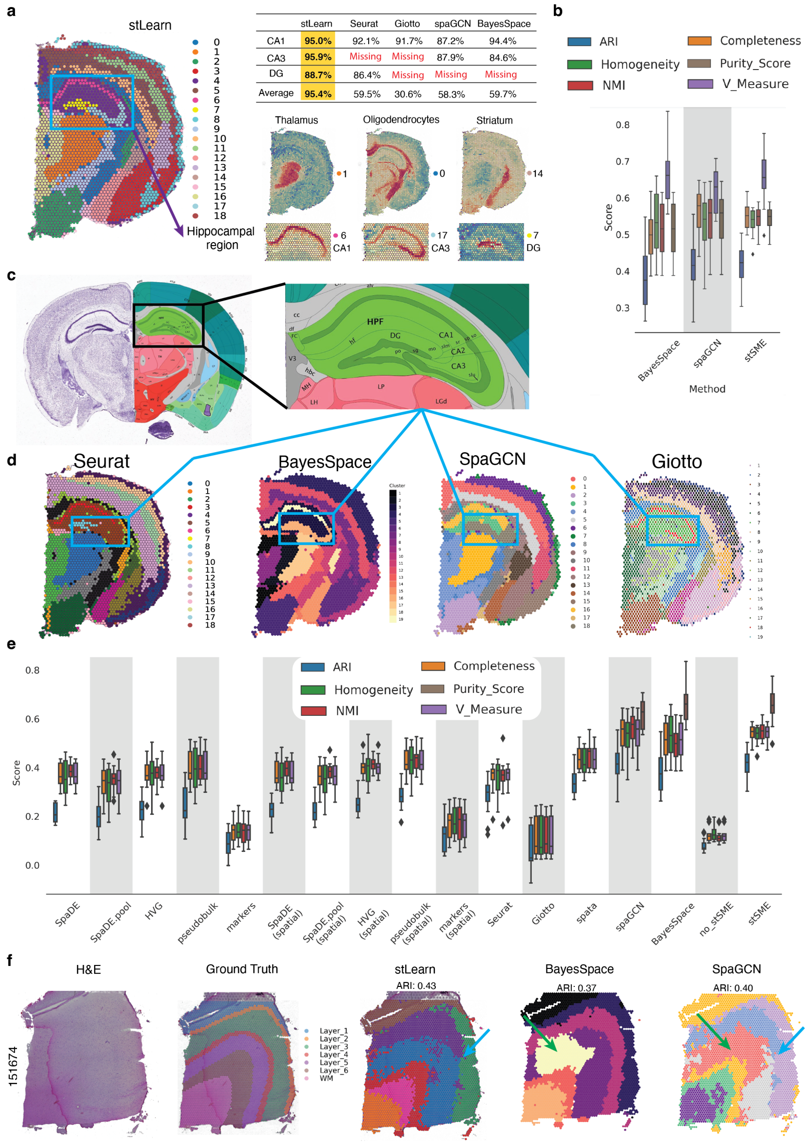

Figure 22. **Assessment of the effects of stSME imputation on clustering results.** **a**, stSME-adjusted Visium data for a coronal section of the adult mouse brain (2,702 spots), showing Louvain clustering results defining 19 distinct clusters. The blue box highlights the hippocampus, where three main sub-regions were resolved, namely cornu ammonis 3 (CA3; cluster 17), CA1/2 (cluster 6), and the dentate gyrus (DG; cluster 7). These three sub-clusters, along with those representing the thalamus (cluster 1), oligodendrocyte distribution (cluster 0; white matter tract proxy), and the striatum (cluster 14), are also shown (*bottom right*; color gradient indicates gene marker expression). The three discrete anatomical (sub-) regions of the hippocampus successfully detected by stLearn were missed, to varying degrees, by Seurat[1], SpaGCN[2], and Giotto[3] (see Table and compare **a** (*top left*) with **d**). To ensure an objective comparison between methods, we used identical input data and constrained the cluster number (resolution) to 18-19 clusters per method. **b**, Comparison of stLearn with BayesSpace and SpaGCN clustering results, using six different assessment metrics (for the mouse brain section containing 2,702 spots). **c**, Mouse brain reference data from the Allen Mouse Brain Atlas used to compare clustering results between analysis methods. Mouse brain H&E image (*left hemisphere*), the corresponding brain anatomical reference regions (*right hemisphere*), and a zoomed-in view of the hippocampus (*right*) are shown. **d**, Comparison of clustering results from four alternative tools, Seurat, BayesSpace, SpaGCN and Giotto. The hippocampus region is marked up by the blue box; all four methods fail to detect one or both of the CA3 and DG regions. **e**, Benchmarking comparisons of clustering results for stSME-adjusted Visium human dorsolateral prefrontal cortex data[4] against other approaches/methods, as listed. Six clustering performance metrics (derived from n=12 sections within this dataset[4]) were used for each comparison, namely ARI, completeness, homogeneity, purity, normalised mutual information (NMI) and V-measure, which combines completeness and homogeneity **f**, Example of clustering results from stLearn, BayesSpace and SpaGCN for the layered structure of the human prefrontal cortex. The corresponding H&E image and ground truth (defined by histological drawings/annotations as in[4]) are also shown.

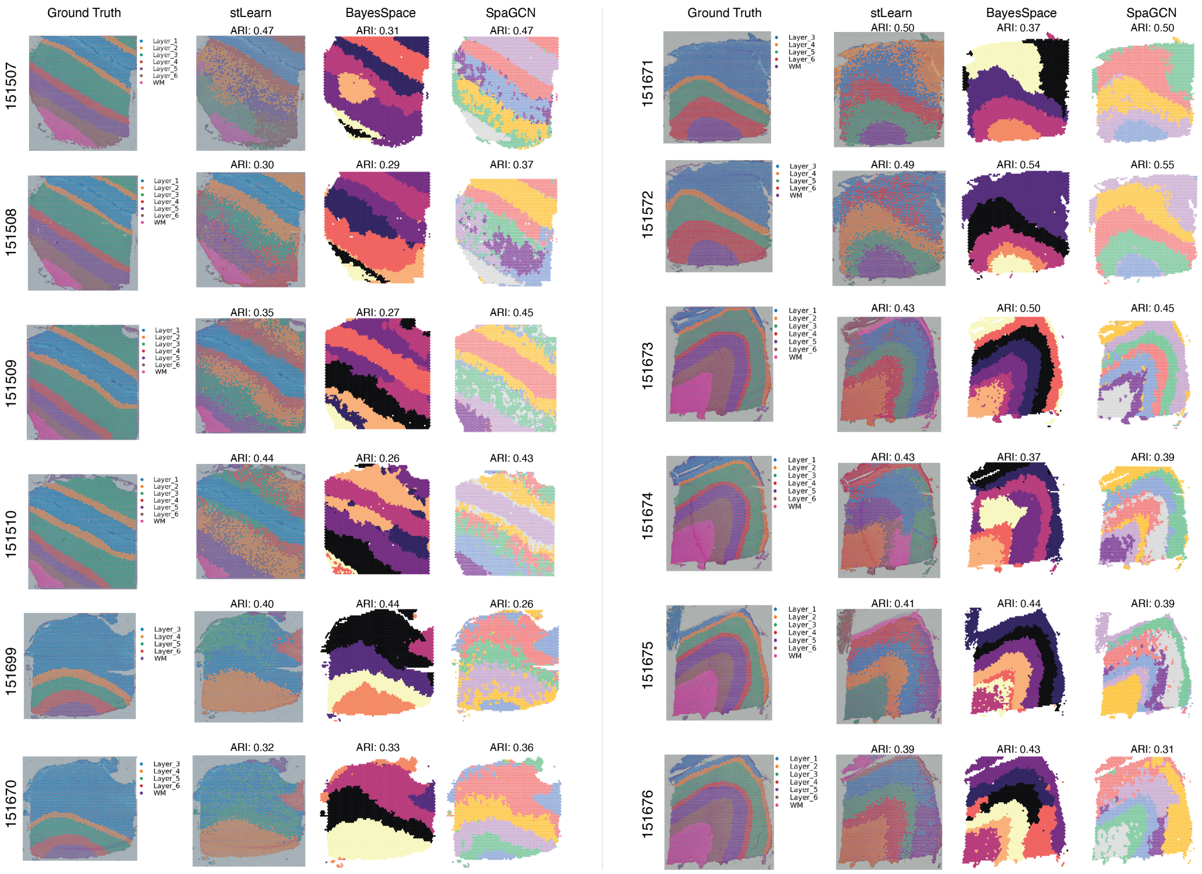

Figure 23. Complete comparison of clustering results analysis between stLearn, BayesSpace and spaGCN for all sections in the Visium human dorsolateral prefrontal cortex dataset. Clustering results against the ground truth (first column) and ARI values are shown for stLearn (second column), BayesSpace (third column) and SpaGCN (fourth column) for each of the n=12 sections available within this dataset[4].

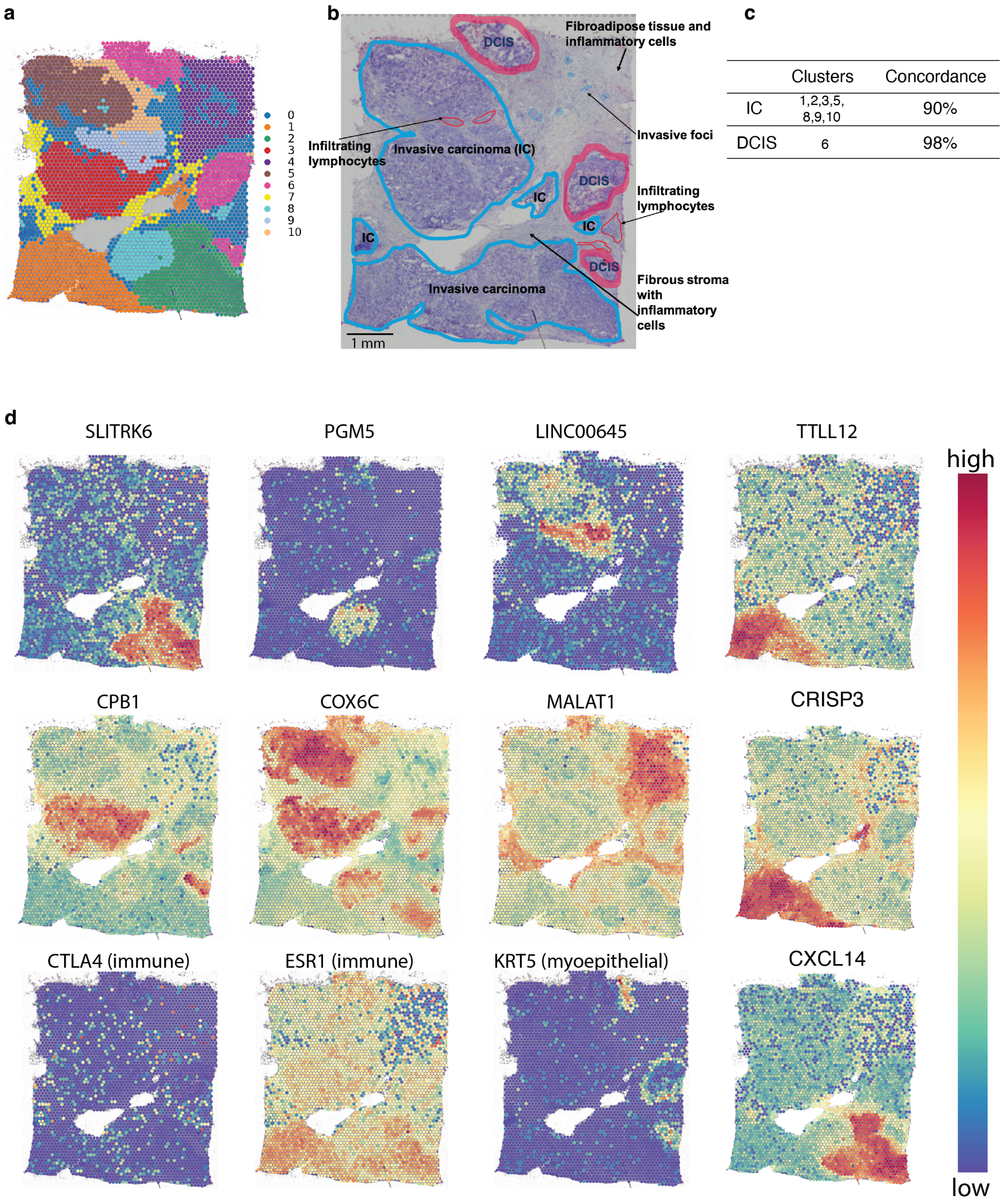

	Clusters	Concordance
IC	1,2,3,5, 8,9,10	90%
DCIS	6	98%

Figure 24. Molecular markers for optimised breast cancer clusters or states. **a**, Unsupervised cell type identification by stLearn which identified 11 clusters. Clusters are labelled from 0 to 10, and spots that belong to the same cluster share the same colour. **b**, Morphological annotation by expert breast cancer pathologists using the high-resolution H&E image. Ductal carcinoma in situ (DCIS) and invasive carcinoma (IC) regions are highlighted in pink and blue, respectively **c**, Comparison of stLearn clustering with expert pathological annotation ($n_{slide} = 1$, $n_{spot} = 3,813$). **d**, Top gene markers identified by stLearn for all IC and DCIS clusters, showing clearly distinct molecular patterns that discriminate within and between these regions. For example, two genes (*CXCL14* and *CRISP3*) can distinguish between two IC states (SMEclust clusters 1 and 2) that correspond to one large pathologist-defined IC region at the bottom of the tissue (compare **b**, bottom blue outline with **d**, right column, bottom two panels)

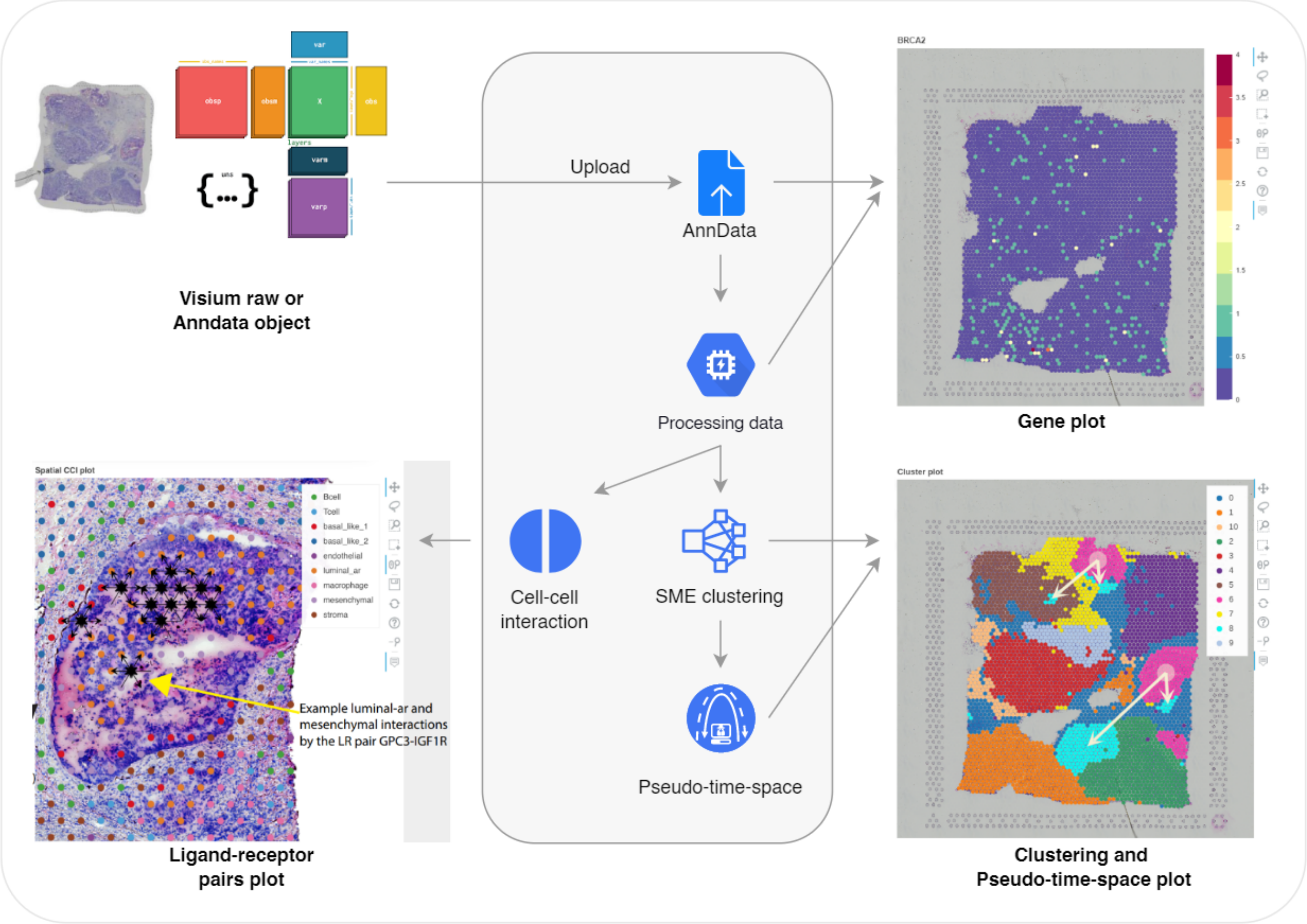

Figure 25. Interactive stLearn (i-stLearn) analysis workflow. The i-stLearn software can support Visium data input in both raw format (gene expression and image data), or AnnData format for other types of spatial transcriptomics data. The i-stLearn workflow starts with loading and pre-processing data, followed by clustering, differential expression analysis, cell-cell interaction analysis, clustering and trajectory inference. First, an upload module in the interactive stLearn app can be used to read the input data within several seconds to a minute. Next, the software provides a processing module to normalise, transform and calculate a nearest neighbour graph using the uploaded data. After that, users can interactively select and visualise gene expression within its spot/cell context on the H&E image. Interactive visualisation functions, like zooming in and out, lasso selection, and saving images are available. These functionalities are especially useful for comparing gene expression and tissue morphology, and they can also be used to annotate tissue regions. The annotation can be saved to an Anndata object. The downstream analysis modules start with stSME clustering (with an option to run stSME normalisation, including image tiling and deep learning feature extraction) to find cell types and spatial organisation. Post-clustering, pseudo-time-space (PSTS) inference can be implemented to find trajectories between clusters and sub-clusters. Users can visualise clusters and trajectories using the clustering plot module. Differential expression analysis module is available for comparing gene expression between clusters. stLearn's comprehensive spatially-constrained two-level permutation (SCTP) analysis module for studying cell-cell interactions (CCIs) starts by finding significant interacting spots and significant interacting ligand-receptor pairs, followed by CCI analysis. In each analysis module, resulting graphics can be saved as png or svg images. Analysis data can be updated and saved in an Anndata object, which can be exported to be re-uploaded later, or for additional downstream computational analysis.

References

1. Stuart, T. *et al.* Comprehensive integration of single-cell data. *Cell* **177**, 1888–1902 (2019).
2. Hu, J. *et al.* SpaGCN: Integrating gene expression, spatial location and histology to identify spatial domains and spatially variable genes by graph convolutional network. *Nat Methods* **18**, 1342–1351 (2021).
3. Dries, R. *et al.* Giotto: a toolbox for integrative analysis and visualization of spatial expression data. *Genome Biol* **22**, 78 (2021).
4. Maynard, K. R. *et al.* Transcriptome-scale spatial gene expression in the human dorsolateral prefrontal cortex. *Nat Neurosci* **24**, 425–436 (2021).

Supplementary Note 1

Pseudotime and Pseudotime-space (PSTS)

1 Extending Pseudo-time-space concepts

We described the pseudo-time-space (PSTS) algorithm in the **Methods** section of the main text. In this supplementary note, we further discuss the concepts underlying PSTS, provide more details about our algorithm from both computational and biological points of view, and also the various settings used in benchmarking comparisons.

Overview and rationale of the PSTS algorithm

We developed an algorithm called pseudo-time-space (PSTS) for spatial trajectory analysis. PSTS is designed to detect biological processes that can be inferred from gradient changes in transcriptional states across a tissue. The algorithm has two main components: pseudo-temporal values derived from gene expression information (temporal) and physical location data of individual spots (spatial) (Fig. S1a-f, formulae 10-12). The inclusion of spatial data provides a significant improvement in trajectory analysis results as it enables the detection of spatial branching processes/phenomena (Fig. S1e; more details below). This allows us, for example, to discover or reconstruct *in vivo* processes within a tissue, such as to understand cellular responses during neuroinflammation, to find the migration and/or differentiation of immature neuronal cells during development, or predict multiple clades of cancer evolution.

Pseudotime and Pseudotime-space (PSTS)

Pseudotime or pseudo-temporal ordering analysis[1] is a popular concept in single-cell RNA sequencing (scRNA-seq) data analysis. Pseudotime was invented to solve the challenge of measuring transcriptional changes as cells carry out biological processes and/or transition into different states[1], particularly in situations where a time-course is not available. In a hypothetical scRNA-seq sample of cells undergoing some progression (e.g. activation, differentiation, or cell division), even if all the cells were captured at exactly the same time, we would still observe the cells to be greatly distributed in terms of their individual progression and/or state. Specifically, some cells would still be at an early stage while others would have moved to more advanced stages of the progression that is being measured; these differences would also be reflected in the cells' transcriptomes[2]. This asynchrony phenomenon therefore suggests a solution to infer changes in cell states and/or progression from only a single snapshot of time. A tracking algorithm could thus be applied to gene expression of all cells within a given snapshot, revealing the variability of gene expression because of the genes' kinetics. To capture this asynchrony process, trajectory analysis methods have been developed using scRNA-seq data; these methods infer cell states based on only one snapshot of sampling time[1,3,4]. In summary, pseudotime is an abstract unit of progression, measuring the transcriptional distance between cells from the start to the end of a given temporal trajectory.

As discussed in the main text, one drawback to scRNA-seq approaches is that anatomical information about a cell's location within the broader tissue is lost, as is context from the local cellular neighbourhood. Trajectory reconstructions are generated under the assumption that all cells of the same cell type were originally physically close to each other (i.e. formed a single cell population, albeit with cellular variability). However, this assumption does not hold if one cell type is in fact distributed across different regions, or where region-specific changes for that cell type may occur; examples of this would include metastatic tumours as well as instances of tissue injury and inflammation. This shortcoming can be solved with spatial transcriptomics, if gene expression information is correctly coupled to cellular distribution data. We therefore created the pseudo-time-space algorithm to reconstruct spatial trajectories that can track (or identify) pseudo-temporal patterns of cell states and/or transitioning in ST datasets.

Branching processes

The theory of branching processes is a mathematical representation that explains the situations in which an object exists in a time before being replaced by one, two or more entities of a similar or different kind[5]. In biology, many dynamic processes have branching characteristics, including cell division, morphogenesis, clonal drug resistance, developmental differentiation, and evolution[6]. The PSTS algorithm is inspired by the theory of branching processes, and we performed a cancer progression case study to demonstrate the utility of PSTS in modelling biological branching processes (Supplementary Note 1). In brief, cancer samples may contain multiple areas of cancerous cells distributed across the tissue. For example, multiple cancerous regions may be found in different ducts in the breast, or in two different regions more generally, and it is useful to know where such regions originated from, as in, whether they share a different or common origin. Cancer relatedness and origins can be modelled in ST data using as input the location of cells to detect branching characteristics, and gene expression to infer cell relatedness, kinetics and/or direction. This allows for the reconstruction of complex spatial trajectories that depict branching processes within the tissue context of the profiled cells, which is a significant advance from being able to generate linear spatio-temporal paths from gene expression data only.

2 Parameter settings for spatial trajectory benchmarking comparisons

PSTS, SpaceFlow, SlingShot, and Monocle3 algorithms were compared using the default parameters defined by each method.

Specifically, for PSTS, we applied the default PCA settings in stLearn, which utilized morphological information from `stSME ResNet50`, with a radius of 50 pixels, and the spot mean expression aggregation option. Neighborhood graphs were subsequently computed with `n_neighbors=25`, based on the stSME adjusted PCA. The analysis using PSTS was conducted in two stages: first, pseudo-time-space values were calculated, selecting `root 1` and setting `reverse` to `True` (the same root selection protocol was also applied for SpaceFlow, SlingShot and Monocle3 runs); secondly, a spatial trajectory was reconstructed using `eps=100`, `threshold_spots=15`, followed by the automated, unsupervised selection of the shortest route from a set of optimal routes - a unique feature to PSTS. For comparative analysis, PSTS values were extracted and compared visually against results from other methods. The complete reproducible code is available at our paper's GitHub site: Shortest path algorithm implementation.

For SpaceFlow, the parameters used for the deep learning model were as per the default settings (available at the SpaceFlow repository): `spatial_regularization_strength=0.1`, `z_dim=50`, `lr=1e-3`, `epochs=1000`, `max_patience=50`, `min_stop=100`, `regularization_acceleration=True`, and `edge_subset_sz=1000000`. All other steps were also implemented as per the default `pseudo_Spatiotemporal_Map` function, with SpaceFlow cluster parameters as `n_neighbors=20` and `resolution=1.0`. The resulting pSM values were extracted and compared with stLearn's PSTS values in finding correlated genes and pathways as described later.

SlingShot was run with the default scRNAseq settings (as described in the Slingshot tutorial. Briefly, we employed PCA for dimensionality reduction and other analysis settings with `thresh = 0.001`, `maxit = 15`, `stretch = 2`, `smoother = 'smooth.spline'`, `shrink.method = 'cosine'`, and `allow.breaks = TRUE`. All potential lineages generated by SlingShot were based on these settings, thus enabling user-defined trajectory selection. We utilized the pseudotime data to determine the trajectory/dynamic pattern in the PCA/UMAP latent space. Slingshot's pseudotime values were again extracted for comparison to PSTS, as detailed above.

For Monocle, we used the default Monocle3 scRNAseq settings (as described in the Monocle 3 tutorial), with PCA selected as the dimensionality reduction method. We used `use_partition = TRUE`, and `close_loop = TRUE`, with the default parameters for partitioning being: `k = 25`, `euclidean_distance_ratio = 1`, `geodesic_distance_ratio = 1/3`, and `prune_graph = TRUE`. Based on these settings, Monocle3 offers an interactive dashboard for root/terminal selection. We chose the damaged site as the root and reversed the pseudotime values. The outcomes were extracted for comparison with PSTS values.

3 Spatial trajectory selection

First we compared the spatial gradient across the whole tissue. Figure S6a illustrates that SpaceFlow, which operates with a neural network incorporating spatial regularization, did generate a spatially smooth, more continuous gradient of pseudotime scores across the tissue; this is reflected in lower semivariance values (0 to 0.05) for SpaceFlow in its variogram compared to stLearn PSTS (0 to 0.135). SpaceFlow was not designed, however, to detect transitioning trajectory paths.

In an unsupervised process, PSTS can identify the shortest (optimal) path from a set of all possible paths (or routes) through the following four automated sequential steps: First, a spatial-PAGA graph is constructed based on the gene expression data and all cluster labels (*step 1*; Fig. S6a). This fully connected graph allows every node to be initially connected to all other nodes. Nodes in the spatial-PAGA graph correspond to unsupervised stLearn clusters, while the edges represent distance measures calculated from both spatial coordinates and gene expression data (Algorithm 1). Next, the cluster with the highest and lowest PSTS score are selected (cluster 1 and 4 for the Visium mouse traumatic brain injury (TBI) ST-seq dataset), and a set of all possible paths (i.e., from the lowest to the highest PSTS score) created based on the spatial-PAGA graph (*step 2*). In the Visium TBI ST-seq dataset, this yielded a total of 170 possible paths to travel from cluster 4 to cluster 1 in the initial graph. The physical spatial distance across the tissue section is then computed for each path, enabling their ranking (*step 3*; Fig. S6b). For the Visium TBI ST-seq dataset, the path with the shortest total spatial distance from cluster 4 to cluster 1 was identified as $4 \rightarrow 2 \rightarrow 3 \rightarrow 1$, with a total distance of approximately 1578 pixels (Fig. S6b). In the final step, the optimal path is highlighted while other connections (graph edges) are trimmed for visualization purposes (*step 4*).

4 Comparative Analysis of PSTS and SpaceFlow pseudotime scores in Downstream Biological Analysis

We found evidence within the Visium mouse TBI ST-seq dataset that the optimised route, as identified by PSTS, produced more biologically meaningful results in the number and relevance of the transition genes and/or pathways detected compared to SpaceFlow. Most notably, this included the ability to detect microglia activation pathways (a process we experimentally validated relative to the damage site, both spatially and temporally). PSTS outperformed SpaceFlow here in detecting microglia-related transition genes, i.e. those with expression values that are significantly associated with pseudotime scores across the

whole tissue section. Specifically, PSTS detected 121 transition genes (which included microglia activation markers such as *C1qa*, *C1qb*, *Tyrobp*, and *Fcer1g*; Fig. S6c) and associated pathways, including the top-ranked microglia pathogen phagocytosis pathway, TYROBP causal network, and oxidative damage/stress (Figure S6d). SpaceFlow results only detected 3 transition genes and no microglia-related pathways in the enrichment analysis.

5 Case study: Spatial trajectory inference in human breast cancer

In the **Results** section of the main text, we report the pseudo-time-space (PSTS) analysis results for microglia activation in a mouse model of traumatic brain injury (3 days post-injury), along with the corresponding experimental validation of the obtained trajectories. In this supplementary note, we further illustrate the concept and potential of PSTS in a second case study where we use a human breast cancer dataset.

Non-invasive ductal carcinoma *in situ* (DCIS) is the earliest-detectable form of breast cancer in mammograms. Although not all detected DCIS will progress to invasive ductal carcinoma (IDC), consistent predictors for future invasiveness remain elusive, thereby complicating clinical management in terms of treatment[7]. Here we therefore applied the PSTS algorithm to a breast cancer ST dataset to explore and demonstrate its usefulness for finding the spatial and transcriptional connections between the DCIS and IDC regions at both the cluster and sub-cluster level.

5.1 Pre-processing:

Human breast cancer spatial data (Block A Section 1, version 1.0.0), publicly available from 10x Genomics, was used for this analysis. This dataset consists of 3,813 spots within the tissue area, and a total of 22,968 genes with a median of 5,394 genes per spot. As per the stLearn vignette for spatial trajectory inference, we applied several standard pre-processing steps, such as filtering low-information genes, normalising counts per spot, log transformation, and scaling each gene to unit variance. This resulted in a working dataset of 3,813 spots and 20,687 genes.

5.2 Clustering and identification of cancerous regions:

We first performed principal component analysis (PCA) using 50 components, before running SMEclust with the spatial-PCA option. This resulted in 11 predicted clusters (Fig. S8a), which we annotated by differential expression analysis (Fig. S9); 9 of these clusters were further split into two or more sub-clusters because they contained spatially-separate spots, resulting in a total of 60 sub-clusters. Based on H&E morphology and differential expression of marker genes, we identified one DCIS cluster comprised of three sub-clusters (sub-clusters 6, 13 and 16; pink in Fig. S8a), and one IDC cluster which comprised of four sub-clusters (sub-clusters 10, 18, 25, 27; sky blue in Fig. S8a). We then also projected the gene expression data into UMAP space (Fig. S8b) and observed that the DCIS and IDC clusters sat next to one another. This further confirms the close relationship of DCIS and IDC clusters at the transcriptional level.

Mascaux et al. previously showed that immune evasion happens in the early stage of squamous lung cancer, prior to tumour invasion[8]. We sought to discover whether breast cancers may behave in a similar manner. From the clustering result of the human breast cancer Visium sample (Fig. S8a,b), we can see that each DCIS region is very close to an IDC sub-cluster. With differential expression analysis, we further observed expression of macrophage gene markers in those IDC sub-clusters, indicative of immune activation here (Fig. S9c). Macrophage gene markers did exist in DCIS sub-clusters, but only at very low levels, providing support for the hypothesis that immune evasion may also occur in early breast cancer, something that can now be further probed experimentally.

Lastly, we originally predicted one DCIS cluster on the basis of gene expression alone; this cluster was then split into three sub-clusters after the incorporation of spatial data (Fig. 3c). Visual analysis of the underlying H&E image also reveals distinct morphological differences between the separate sub-clusters. Specifically, the top DCIS sub-cluster does not contain any visible cancer cells, while the middle DCIS sub-cluster contains a visible population of invasive cancerous cells within the DCIS lesion (Fig. 3e). This suggests that the different sub-clusters represent different stages of cancer cell invasion, which we further explored using spatial trajectory inference with PSTS algorithm (see below).

5.3 Implementation of spatial trajectory reconstruction:

The first steps of spatial trajectory reconstruction proceed as for any other scRNA-seq dataset, that is, incorporating gene expression information only, and making use of the broad clusters rather than the spatially-separated sub-clusters described above. Next, we calculated PSTS values for the entire breast cancer sample (PSTS values are calculated based on gene expression data and spatial distance; see Methods), with the DCIS cluster set as the root. We then built a raw spatial-PAGA graph, which provides a first general picture of gene expression-based cluster relationships across the cancer tissue. Here, each broad cluster is represented as a single node in the graph; only nodes with connected edges are used to reconstruct the spatial trajectories in subsequent PSTS steps. The raw spatial-PAGA graph is then used to construct a final spatial-PAGA graph, with cluster nodes split into one or more further nodes separated by their spatial location if they represent clusters with multiple

sub-clusters (such as the three DCIS and four IDC sub-clusters described above). Edge weights were set to equal d_{PTS}. The calculated spatial-PAGA graph can then be projected onto the tissue array image for visualisation.

Although it is possible to calculate a spatial trajectory across an entire tissue section with no prior biological knowledge, our spatial trajectory algorithm works best with a subset of clusters that are predicted to share some biological connection. For example, as we were interested here in modelling DCIS-IDC progression, we select the DCIS (pink) and IDC (sky blue) clusters as the root and endpoints of the trajectory, respectively. Therefore, although the spatial-PAGA graph was constructed for the entire tissue section, we observed that the DCIS and IDC lesions are directly connected in the graph. We then subset the graph to include only those nodes from DCIS to IDC clusters, and the edges between them. Finally, we convert the spatial-PAGA graph to a directed spatial-PAGA graph, based on the difference of PSTS values between clusters; this graph is a directed bipartite graph. In situations where we either do not know the precise original root cell, or where this cell may no longer exists due to cell division, we include a hypothetical pseudo-root at the start of the trajectory, resulting in an arborescence with the order: pseudo-root → DCIS → IDC. Nodes represent the pseudo-root, the DCIS and IDC sub-clusters, while edges represent d_{PTS} values.

Previous studies have shown the pattern of cancer development to be affected by spatial proximity[9,10]. Progression, invasion and/or differentiation of cells generally occur here in the regions closest to themselves. Therefore, the fully-connected arborescence calculated in the previous step is most likely over-connected, containing connections that are not necessarily always biologically meaningful. Thus, in the final step, we optimise the graph by finding the shortest path (minimising d_{PTS} distance) between the root and end nodes by applying the Minimum Spanning Arborescence to the graph. This results in the final spatial trajectory predictions between DCIS and IDC, with three clades that represent cancer progression from DCIS to IDC (Fig. S8d).

Characterisation of spatial trajectories and prediction of trajectory-associated marker genes

The spatial trajectories predicted by stLearn can be further characterised, and evidence that they are indeed distinct lineages can be assessed as below.

Phenotype (by H&E image): We obtained expert pathological annotations of the H&E image from three independent pathologists. The tissue morphology shows a distinction between two clades of the spatial trajectory (see Supplementary Note 1.5 and Fig. S25b). Specifically, the top DCIS sub-cluster (clade 1) does not contain any visible cancer cells while the middle DCIS sub-cluster (clade 2) contains a visible population of invasive cancerous cells within the DCIS lesion (Fig. 3e, Fig. S8 and S24b). IDC progression can typically be monitored via an H&E image. In our dataset, the clade 2 IDC sub-cluster shows phenotypic characteristics of invasiveness while the clade 1 IDC sub-cluster does not (Fig. 3e and Fig. S8). However, both sub-clusters express gene markers associated with invasive cancer and immune responses (described below).

Transition markers (by gene expression): We developed an analysis in stLearn to first find transition markers (genes that are significantly associated with PSTS scores), and to then compare these transition markers across. For the cancer lineages, by comparing the two clades, we found unique signature genes for each. Examples are shown in Fig. S11 and S12. Previous studies have shown the pattern of cancer development to be affected by spatial proximity[9,10]. We here performed transition marker analysis to identify those genes that either increase or decrease their expression along the trajectory/trajectories of each clade (Fig. S11). The upregulated genes, which increase their expression along the DCIS-to-IDC trajectory, included several immune cell markers for macrophages ($Cd74$, $Apoe$) and more generalised immune response genes ($C3$, HLA-Family genes, $Iglc2$, etc.). Downregulated genes, which decrease in expression along the trajectory, also revealed some cancer progression markers such as Mgp and Dsp. stLearn includes a function to also distinguish between transition markers of different clades (Fig. S11 and S12). As a result, we found some very specific markers that may relate to specific stages of cancer progression like $Ier3$ for clade 2, and $Ifi6$ for clade 3 (Fig. S12).

Transcriptional states (by gene expression): We show in Fig. S11d that the sub-cluster members of the two branches have different distributions of pseudotime scores, thus reflecting different transcriptional states. These findings again highlight the significant potential of our PSTS algorithm, advancing the ability to predict invasion potential at the molecular level in this specific setting, i.e. prior to the emergence of visible evidence of cancer invasion.

Optimising weighting factor ω in the PSTS algorithm

In the **Method** section, we describe the parameter optimisation for PSTS, which includes the weighting factor ω. We disentangle gene expression and spatial components of a trajectory via our ω optimisation step. The ω analysis shows and explains the contribution of these two aspects towards the reconstruction of a spatial trajectory. Recall that ω can take any value between 0 and 1, and reflects the desired contribution of gene expression ($\omega = 1$) and physical distance $\omega = 0$ for calculating d_{PTS} and the graph optimisation step. The choice of the optimal ω value is determined by comparing graphs where ω changes from 0 to 1, with two references graphs constructed that either do, or do not, use spatial information (Fig. S10). For optimisation of ω, we calculated the spectral distance between the G_ω with two reference graph G_0 (spatial, A1) and G_1 (gene expression,

A2). We then check whether their ratio is near 1 (balanced) or not by the dissimilarity score (Fig. S10a). Here, a biological viewpoint is provided as to why we perform this computational step. Taking our DCIS-IDC case study as the example, we generated spatial trajectories with the two reference graphs using either gene expression ($\omega = 1$) or spatial distance ($\omega = 0$) alone. The 'spatial distance-only' analysis with a PSTS $G_{\omega=0}$ graph appeared to represent the relationship between DCIS and IDC sub-clusters (Fig. S10a). In contrast, when $G_{\omega=1}$ (Fig. S10b), all IDC sub-clusters are connected to only a single DCIS sub-cluster, conflicting with the finding by de Bruin et al.[9] who found evidence for spatially branched evolution of cancer sub-clones. The ω optimisation step indicates $\omega = 0.46$ to be the optimal parameter value in this case (Fig. S10b), providing a balance between the contribution of spatial data and gene expression data that fitted to the spatial relationship.

6 Case study: Spatial trajectory inference in neurodevelopment using single-cell resolution sci-Space data[11])

For this case study, we applied PSTS to neuronal migration dynamics in development[11]. We aimed to detect and localise the coordinated processes of neuronal differentiation and migration, from radial glia to neurons. In the original paper, the authors run a pseudotime algorithm (Monocle3[12]) to model the pseudotemporal spatial trajectories[13–16]. Their analysis shows the trend in the pallium where immature neurons migrate and differentiate radially outward, leading to the inside-out development of the cortical layers. However, the Monocle3 trajectory analysis results were not mapped back spatially to the tissue. By adding spatial information, we expected that PSTS would be able to connect nearby cells into complex trajectories that reflect developmental neurogenesis and neuronal migration.

We first pre-processed the data and then subsetted the brain regions using both the raw and metadata of the sci-Space dataset. The diffusion pseudotime algorithm (DPT) was then applied to compute pseudotime values. To reconstruct the spatial dynamics, we separated the dataset into multiple samples, which correspond to the slide ID of the original experiment. For each sample, we applied PSTS to model the spatio-temporal migration from radial glia to neuronal cell types. With PSTS, we indeed successfully reproduced the result from the sci-Space paper for modelling the neuronal migration dynamics (Fig. 3a,b; Fig. S7). Moreover, based on the spatial information, PSTS was able to extend by reconstructing multiple branching processes, represented by visible trajectories, that show the change from radial glia to neurons[16]. Beyond these results, this case study also demonstrates the broad application of stLearn and specifically PSTS to spatial data at single-cell resolution, and also data without imaging information.

Taken together, we conclude therefore that our PSTS algorithm can reveal spatial trajectories that have relevant (bio)medical and/or diagnostic applications, as demonstrated in the context of neural injury and associated inflammation, developmental processes, as well as cancer progression (see main text).

References

1. Trapnell, C. *et al.* The dynamics and regulators of cell fate decisions are revealed by pseudotemporal ordering of single cells. *Nat. biotechnology* **32**, 381 (2014).
2. Nguyen, Q. H. *et al.* Single-cell RNA-seq of human induced pluripotent stem cells reveals cellular heterogeneity and cell state transitions between subpopulations. *Genome Res* **28**, 1053–1066 (2018).
3. Saelens, W., Cannoodt, R., Todorov, H. & Saeys, Y. A comparison of single-cell trajectory inference methods. *Nat. Biotechnol.* **37**, 547–554 (2019).
4. Wolf, F. A. *et al.* PAGA: graph abstraction reconciles clustering with trajectory inference through a topology preserving map of single cells. *Genome biology* **20**, 59 (2019).
5. Harris, T. E. *The theory of branching process* (Rand Corporation, 1964).
6. Axelrod, D. & Kimmel, M. *Branching processes in biology* (Springer-Verlag, 2015).
7. Dettogni, R. S. *et al.* Potential biomarkers of ductal carcinoma in situ progression. *BMC cancer* **20**, 119 (2020).
8. Mascaux, C. *et al.* Immune evasion before tumour invasion in early lung squamous carcinogenesis. *Nature* **571**, 570–575 (2019).
9. de Bruin, E. C. *et al.* Spatial and temporal diversity in genomic instability processes defines lung cancer evolution. *Science* **346**, 251–256 (2014).
10. Wang, D. *et al.* Multiregion sequencing reveals the genetic heterogeneity and evolutionary history of osteosarcoma and matched pulmonary metastases. *Cancer research* **79**, 7–20 (2019).
11. Srivatsan, S. R. *et al.* Embryo-scale, single-cell spatial transcriptomics. *Science* **373**, 111–117 (2021).
12. Cao, J. *et al.* The single-cell transcriptional landscape of mammalian organogenesis. *Nature* **566**, 496–502 (2019).
13. Nadarajah, B., Brunstrom, J. E., Grutzendler, J., Wong, R. O. & Pearlman, A. L. Two modes of radial migration in early development of the cerebral cortex. *Nat. neuroscience* **4**, 143–150 (2001).
14. Turrero García, M. & Harwell, C. C. Radial glia in the ventral telencephalon. *FEBS letters* **591**, 3942–3959 (2017).
15. Tan, S.-S., Valcanis, H., Kalloniatis, M. & Harvey, A. Cellular dispersion patterns and phenotypes in the developing mouse superior colliculus. *Dev. biology* **241**, 117–131 (2002).
16. Watanabe, Y., Sakuma, C. & Yaginuma, H. Dispersing movement of tangential neuronal migration in superficial layers of the developing chick optic tectum. *Dev. biology* **437**, 131–139 (2018).

Supplementary Note 2

stLearn spatial cell-cell interaction analyses

1 stLearn spatial cell-cell interaction analyses

We present a spatial method to detect cell-cell interaction across the whole tissue using both spatial location and gene expression information. The physical distance is an important constraint parameter to detect cell-cell interactions as nearby cells are more likely to interact than distant cells[1–3], and ligand-receptor based interactions typically operate within a 0 to 200 μm distance[4,5]. stLearn calculates interactions between neighbouring spots or within spots (if a spot has more than one cell), thereby considering both juxtacrine, paracrine and endocrine LR interaction mechanisms.

2 stLearn spatially-constrained two-level permutation (SCTP)

SCP analysis statistically screens for all known LR pairs and cell type combinations across a tissue section, thereby controlling for false discovery. By adding spatial information to increase the accuracy of statistical predictions of cellular interactions via one or all known ligand-receptor (LR) pair(s) across the whole tissue section, we present a significant advance in the emerging CCI analysis area (Fig. 1d, 4-5, Fig. S14-S18). Three types of cellular cross-talk are considered: autocrine (by the same cell), juxtacrine (via cell-cell contact) and paracrine (between non-adjacent neighbouring cells). All of these interactions operate most strongly within a range of 0 to 200 μm[4,5]. stLearn's SCTP analysis detects non-random patterns of LR expression within spatial neighbourhood constraints. In the case of Visium data, we also developed a 'within-spot' (55 μm capture area) analysis to detect interactions between 1-9 cells across thousands of spots within a tissue section. By analysing the tissue area, rather than one cell, the within-spot SCTP analysis mode does not require single-cell resolution. stLearn SCTP also tests for paracrine interactions, between cells from neighbouring spots (two spots spanning 155 μm distance). stLearn's between-spot mode can also be directly applied to single-cell resolution datatypes. Optionally, for large datasets consisting of tens-of-thousands to millions of cells, stLearn SCTP can scale to incorporate such massive data by binning the tissue section into grids of a fixed size and taking the average transcriptional profile across cells within the bin prior to performing the CCI analysis. stLearn SCTP can thus be applied to any spatial transcriptomics/proteomics technology, and will scale to increasingly large cell sizes in a single spatial-omics assay. We found that stLearn SCTP outperforms existing CCI methods, especially with regard to biological plausibility through the prediction of individual CCI events within snapshot spatial transcriptomics data. We find this increased resolution, achieved by incorporating spatial information, addresses the key issue of high false positive rates in other CCI methods that do not consider spatial location, or do so less optimally (Fig. 5). Furthermore, due to the cell type information randomisation step (Fig. 4a), stLearn SCTP analysis can take into consideration cases where spatial profiles are mixtures of cell types (as is relevant in e.g. Visium data), without which other methods make many false positive predictions (Fig. 5).

stLearn SCTP allows integration of cell type information, based on the observation that greater cell type diversity is positively correlated with increased likelihood of cell-cell interactions[1,6,7]. The relationship between cell type diversity and cell-cell interaction is, nevertheless, dependent on biological contexts. For example, cancer-immune cell interactions are different to neuron-glia interactions. Therefore, it is optional in our stLearn SCTP analysis to introduce an additional heterogeneity weighting to each of the LR co-expression scores (Fig. 4a). By deconvolution (e.g. using RCTD[8]) or cell annotation (e.g. by label transfer[9]) we predict either one or a mixture of cell types for a cell/spot/bin (see Methods section). Mapping LR co-expression weighted by cell diversity scores to the original tissue displays those spots with high LR co-expression and cell type diversity, while statistical tests (described below, and also Methods section) detect significant cell-cell interaction events (Fig. 4a). To assess the contribution of spatial and cell type diversity, we developed a simulation approach, taking into account cell type-specific gene expression distribution, zero proportions, cell type proportions, and spatial cell communities with differential co-localisation or exclusion of cell types across the tissue (Methods section; Fig. 5b).

Two types of statistical tests were developed for each LR pair, both based on the establishment of a robust, randomised background signal as the null distribution, which consists of $2*\sqrt{k}$ genes (default of k is 1000) in the spatial dataset that are in the same expression range as the query LR pair, and not within the known 2,200 LR pairs (curated by the NATMI[2] database). To select each of the $2*\sqrt{k}$ candidate genes for a given query gene (either the ligand or receptor), we select genes with the most similar expression distribution based on defined quantiles, deliberately oversampling at higher quantiles to account for data sparsity (see Methods and Fig. 4a). Genes are then randomly paired from those most similar in expression to either the ligand or the receptor, generating k random gene-gene pairs. To assess whether a robust number of random pairs has been obtained in a dataset-specific manner, stLearn SCTP implements diagnostic plots that automatically examine the relationship between LR expression level and LR rank based on the number of significant spots; this graph-based approach allows the user to see whether the LR expression level has been effectively controlled by this method (Fig. S14j-k; S15e-f).

Statistical testing for spatial SCTP scores is performed based on the above-detailed random pairing of 1000 (default parameter, can increase to 10,000 or more) gene-gene pairs with similar expression ranges to establish the empirical null distribution for any LR pair. A background distribution per spot and LR pair is established by calculating the LR_{score} (or optional LR'_{score}) for each random pair across all spots/cells. Each spot has k 1000 random scores for each LR. P-values for each spot and LR pair ($p_{s,LR}$) are then calculated as the proportion of the background scores (b_i) across k random pairs that had

a score greater than the LR_{score}.

To determine significant cell type-cell type interactions via each LR pair of interest, stLearn SCTP introduces an additional permutation test. This analysis starts with the results of significant spots and significant LRs defined as above. For each LR pair, we calculate a significant cell type count matrix CCI_{LR} of shape $n_c \times n_c$, where n_c is the number of all predicted cell types (either discrete value with one cell type per spot, or a continuous probability with multiple cell types per spot). For two given cell types x and y, we counted significant spots for row x (signal-sending cell types; ligand expressing) and column of y (signal-receiving cell types, receptor expressing). We then permuted the cell type information associated with each spot k times and re-calculate CCI_{LR} with the cell type information permuted. The significant p-value for two cell types to interact via a given LR pair is the proportion of permutation runs where the CCI_{LR} count was higher than the background count (see Fig. 4a and see Methods section).

3 Applications of stLearn SCTP to test and validate cellular interactions in skin and breast cancer tissues

CCI findings from basal cell carcinoma, validated by RNAscope imaging: In addition to testing for *IL34* and *CSF1R* interactions in BCC (Fig. S17, validated in Fig. S18), we also screened through all of the 550 LR pairs that had detectable gene expression in this dataset. In doing so, we detected a number of other potential interacting LR pairs (Fig. S17), with the most significant pair being *COL1A1-DDR1*. Furthermore, within this dataset, we also compared the stLearn approach, that is, generating a random background for gene-gene co-expression (detailed above, and Methods) to a naïve approach which does not consider the expression levels of the query ligand and receptor genes and/or their spatial enrichment (Fig. S17). We find that the use of an stLearn SCTP background dramatically reduces the correlation between LR significance and gene expression levels compared to when using the naïve background. This is especially true for abundant pairs. For example, the LR pair *COL1A1-DDR1* was considered to have a CCI event for almost every single spot when using the naïve background, but much fewer significant interaction events (hotspots) were predicted when using the stLearn SCTP. The background distribution generated by stLearn adapts the output here to much better match the LR co-expression level when examining individual spots (Fig. S17).

Unbiased detection of interacting cell types and ligand-receptor pairs in breast cancer: A reference breast cancer dataset[10] was used to annotate ST spots with cell type information by label transfer approach[9]. Each spot was allowed to represent more than one cell type here to account for the multi-cell nature of Visium data. We then tested the significant interactions for each of the 750 LR pairs detected in this dataset (background based on 10,000 random gene-gene pairs, with Bonferroni-Hochberg adjusted p-value for each spot). From this global unbiased analysis, we found one of the top interacting ligand-receptor (LR) pairs to be *GPC3-IGF1R* (Fig. 4h-j; Fig. S15b). While *GPC3* and *IGF1R* were expressed across the tissue section (Fig. S15a), stLearn SCTP found significant co-expression hotspots for this LR pair (Fig. S15a), with no bias towards either gene expression level, gene expression frequency, and/or cell type abundance (Fig. S15a, c-f). Comparison of stLearn SCTP results with independent expert pathologist annotation of the H&E section further showed that the spatial localisation of significant *GPC3-IGF1R* spots correlated strongly with ductal carcinoma in situ (DCIS) regions of the breast cancer (Fig. S15a). When also considering cell type information, we found that *GPC3-IGF1R* interactions were most significant between cells expressing luminal androgen receptor (luminal-ar) within DCIS regions and the mesenchymal breast cancer cells surrounding these (Fig. 4h-j). To assess the likely biological process regulated by the predicted CCI events for the LR pairs, we lastly performed GO enrichment analysis for the top 100 LR with the highest number of significant spots (Fig. S15g). These LR pairs showed strong enrichment of known biological processes mediated by cell-cell signalling, including a significant enrichment for the *ERK1/2* cascade, which is a key signalling pathway in breast cancer progression.

4 SCTP minimises false positive detection of interactions

In both the above, we have shown that stLearn SCTP minimises false positive detection of interactions through comprehensive testing of thousands of ligand-receptor pairs, tens of cell types and across different biological systems from normal development to injured and/or diseased tissues, as presented in this paper. The increased accuracy is achieved by a stringently established null distribution of expression across the tissue, which is the basis for improved accuracy of the permutation and negative binomial tests. Although the statistical test helped reduce the number of false positive spots, we still did see some false positive detection when testing for a negative control scenario, using highly abundant housekeeping gene-gene pairs (i.e. artificial pairs of genes that are not ligand-receptor pairs). We found that 131 out of 3,813 (3.44%) of *GAPDH-ACTB* co-expressing spots remained significant (Fig. S**??**a-d). However, as described above, other methods resulted in much higher false-positive interactions (Fig. 5c-f). To address this inherent limitation in all CCI analyses, we demonstrate that additional GO enrichment testing can be performed based on differentially expressed genes identified, comparing spots with significant LR CCI events

against the remaining spots without the interaction (Fig. S16h-i). For example, in the case of *IL34-CSF1R* interactions[11] within the breast cancer tissue, results from differential gene expression analyses and subsequent GO enrichment testing suggests a strong immune responses in those spots with significant interactions (Fig. S16e-g, i). This finding is supported by independent annotations from expert pathologists, which marked up the same tissue areas as having immune cell presence/infiltration (Fig. S16g). For the *GAPDH-ACTB* pair (negative control scenario), we found much less significant and/or selective enrichment, with most GO terms relating cell metabolism and structural maintenance rather than LR signalling (Fig. S16h-i). Such downstream analysis can thus further help to identify false positives.

5 stLearn's SCTP analysis is scalable to larger datasets and applicable to single-cell resolution spatial technologies

As already alluded to, the SCTP algorithm can be universally applied to other types of spatial data that have LR expression and neighbourhood information. We corroborated this by running SCTP in 'between-spot' mode on a publicly available SeqFish+ dataset from the mouse brain[12] to detect the interactions between neighbouring spots (one cell now being equivalent to one spot). This can be done using either the default mode or, alternatively, a grid-mode where the tissue section is split into n tissue areas/bins (Fig. S14c,g). We visualised the cell distributions with colour representing the cell types according to the paper (Fig. S14a) and derived potentially interacting cells within the tissue (Fig. S14b-g). CCI results were consistent with those reported in the paper, but notably, our stLearn SCTP analysis also identified LR targets not reported in the original paper (which performed CCI analysis using methods that do not consider spatial information). For example, we identify *Gas6* and *Axl* as the most significant top interacting LR pair between ependymal cells, neural stem cells and oligodendrocytes within the subventricular zone (Fig. 4c-d).

Notably, the stLearn SCTP analysis pipeline does not require single-cell resolution because we can test for local co-expression between spots/bins/cells as evidence for paracrine LR interactions. We are also able to find enrichment (LR co-expression) within one spot/bin, which would include both autocrine and juxtacrine LR interactions, and/or local paracrine interactions between cells. To account for multiple cell types being present within the same spot/bin, stLearn SCTP uses a probabilistic mixture of cell types for each spot/bin, as inferred from the transcriptomic profile. Cell type probabilities are derived here from Seurat's label transferring method[13] and RCTD deconvolution[8], and the results are used for the optional cell type diversity component of LR_{score} calculations as well as for the cell type permutation. As an example, to evaluate the effect of having lower-resolution data, we applied a strategy to bin the single-cell seqFISH+ data, and found highly consistent results (Fig. S14b, f-g). Specifically, stLearn SCTP results in grid-mode showed locations and significant LR interactions that were consistent with the single-cell seqFISH+ mode. The reduced resolution did appear to detect fewer significant interactions at cell type level (Fig. S14e *vs.* f), but this is expected.

Of note, the stLearn SCTP grid-mode is fast and can be applied to datasets with thousands to millions of cells. To assess scalability of the stLearn SCTP pipeline, we analysed a much bigger single-cell spatial dataset (Slide-seq, with 47,573 cells)[14], (Fig. 4e-g, Fig. S14h-k). Similar to the analysis of seqFISH+ data, we found that the grid-mode generated consistent results with the single-cell mode, and also that the grid-mode option can be universally applied to bigger datasets. The number of grids could be as high as hundreds of thousands of grids, which would be equivalent to millions of cells. The grid-mode does reduce resolution (Fig. S14), but also brings advantages in terms of reducing the effect of outliers, those with highly abundant expression or, conversely, with technical dropouts (Fig. S14j,k). These beneficial effects come from the pooling of multiple neighbour single cells into one grid. Overall, the stLearn SCTP analysis pipeline, with both single-cell and grid-mode, can thus be scaled up to very large datasets and/or data at different cellular resolutions.

6 SCTP output evaluations

For experimental validation, we generated both Visium ST-seq and RNAscope single-molecule *in situ* hybridisation data for adjacent tissue sections of a basal cell carcinoma (BCC) sample. The RNAscope data detected local co-expression of ligands and receptors predicted by cell-cell interaction (CCI) analysis, validating our results in the BCC sample (Fig. S18). We also evaluated stLearn-predicted CCI results in the breast cancer dataset. CCI spots predicted with significant *IL34-CSF1R* interactions also had a distinct enrichment of downstream immune response pathways (Fig. S16). Consistent to previous studies, independent annotation by professional pathologists, confirmed that these spots were located at the immune interface and/or inflamed regions (Fig. S16g).

In summary, SCTP can be applied to multiple technological platforms. We tested two different types of spatial data, namely sub-cellular resolution Slide-seq[14], and single-cell resolution imaging-based data seqFISH+ data[12] (Fig. S14). Our results here were consistent with those reported in the original paper but also identified additional LR pairs at specific tissue regions, for example *Apoe-Lrp1* and *Gas6-Axl* in Slideseq and seqFISH+ data, respectively.

7 stLearn SCTP benchmarking comparisons

To benchmark the stLearn SCTP analysis pipeline against eight other common CCI methods, we thoroughly compared stLearn's SCTP outputs with those from Squidpy[15], CellChat[16], cellphoneDB[17], NATMI[2], SingleCellSignalR[18], NCEM[19], SpaTalk[20] and spaOTsc[21]. For this benchmarking, we first developed and used a simulated spatial dataset to assess false discovery of cells/spots that are located outside the plausible physical interaction distance, and to evaluate the contributions of spatial distance and cell type heterogeneity to CCI results. We did so by examining the interaction of two cell types A and B (Fig. 5b-d, and Methods). The simulation approach allowed us to generate *in silico* tissues, using interpretable parameters, with expression values preserving cell type signatures, and with the spatial distribution of cell types controlling for colocalisation and exclusion. We found highly accurate predictions for interactions between cell types A-B from the stLearn SCTP pipeline while all other methods, that is, Squidpy, CellPhoneDB, CellChat, NATMI, SingleCellSignalR, NCEM, SpaTalk, spaOTsc predicted multiple interactions for cell types A and B with other cell types, proving the problem of false positive detection that these methods face (Fig. 5d). We then also used the publicly available Visium human breast cancer dataset[22] for further independent testing (Fig. 5e-l). Without spatial information, we consistently found significant interacting LR pairs from distant clusters, suggesting likely false positive detection because the two clusters are located in different microenvironments and/or regions of the tissue section (Fig. 5e,f); these interactions are unlikely to occur as they are well outside of the expected physical distance range of 200 μm[4,5]. In sharp contrast, and consistent with the latter premise (and also the simulation results), stLearn SCTP only detected significant interactions between proximal spots. Thus, by taking into account between- and within-spot interactions, we show much more accurate signals at spot level.

References

1. Schapiro, D. *et al.* histoCAT: analysis of cell phenotypes and interactions in multiplex image cytometry data. *Nat. methods* **14**, 873 (2017).
2. Hou, R., Denisenko, E., Ong, H. T., Ramilowski, J. A. & Forrest, A. R. R. Predicting cell-to-cell communication networks using NATMI. *Nat Commun* **11**, 5011 (2020).
3. Niethamer, T. K. *et al.* Defining the role of pulmonary endothelial cell heterogeneity in the response to acute lung injury. *Elife* **9** (2020).
4. Momiji, H. *et al.* Disentangling juxtacrine from paracrine signalling in dynamic tissue. *PLoS Comput. Biol* **15**, e1007030 (2019).
5. Longo, S. K., Guo, M. G., Ji, A. L. & Khavari, P. A. Integrating single-cell and spatial transcriptomics to elucidate intercellular tissue dynamics. *Nat Rev Genet.* **22**, 627–644 (2021).
6. Rieckmann, J. C. *et al.* Social network architecture of human immune cells unveiled by quantitative proteomics. *Nat. Immunol.* **18**, 583–593 (2017).
7. Eng, C. L. *et al.* Transcriptome-scale super-resolved imaging in tissues by RNA seqFISH. *Nature* **568**, 235–239 (2019).
8. Cable, D. M. *et al.* Robust decomposition of cell type mixtures in spatial transcriptomics. *Nat. Biotechnol.* 1–10 (2021).
9. Stuart, T. *et al.* Comprehensive Integration of Single-Cell Data. *Cell* **177**, 1888–1902 (2019).
10. Karaayvaz, M. *et al.* Unravelling subclonal heterogeneity and aggressive disease states in TNBC through single-cell rna-seq. *Nat. communications* **9**, 1–10 (2018).
11. Lin, W. *et al.* Function of CSF1 and IL34 in Macrophage Homeostasis, Inflammation, and Cancer. *Front Immunol* **10**, 2019 (2019).
12. Eng, C., Lawson, M. & Zhu, Q. Transcriptome-scale super-resolved imaging in tissues by RNA seqFISH+. *Nature* **568**, 235–239 (2019).
13. Stuart, T. *et al.* Comprehensive integration of single-cell data. *Cell* **177**, 1888–1902 (2019).
14. Rodriques, S. G. *et al.* Slide-seq: A scalable technology for measuring genome-wide expression at high spatial resolution. *Science* **363**, 1463–1467 (2019).
15. Palla, G. *et al.* Squidpy: a scalable framework for spatial single cell analysis. *bioRxiv* DOI: 10.1101/2021.02.19.431994 (2021). https://www.biorxiv.org/content/early/2021/04/29/2021.02.19.431994.full.pdf.
16. Jin, S. *et al.* Inference and analysis of cell-cell communication using CellChat. *Nat Commun* **12**, 1088 (2021).
17. Efremova, M., Vento-Tormo, M., Teichmann, S. A. & Vento-Tormo, R. CellPhoneDB: inferring cell–cell communication from combined expression of multi-subunit ligand–receptor complexes. *Nat. Protoc.* **15**, 1484–1506 (2020).
18. Cabello-Aguilar, S. *et al.* SingleCellSignalR: inference of intercellular networks from single-cell transcriptomics. *Nucleic Acids Res* **48**, e55 (2020).
19. Fischer, D. S., Schaar, A. C. & Theis, F. J. Learning cell communication from spatial graphs of cells. *BioRxiv* 2021–07 (2021).
20. Shao, X. *et al.* Knowledge-graph-based cell-cell communication inference for spatially resolved transcriptomic data with spatalk. *Nat. Commun.* **13**, 4429 (2022).
21. Cang, Z. & Nie, Q. Inferring spatial and signaling relationships between cells from single cell transcriptomic data. *Nat. communications* **11**, 2084 (2020).
22. 10x Genomics. Human breast cancer (block a section 1), spatial gene expression dataset by space ranger 1.1.0 (2020).

Supplementary Note 3

stLearn stSME-based imputation analyses

1 stSME imputation reduces technical variation and increases spatial detection coverage

stSME corrects for technical variation in spatial sequencing data by using information from reference spots, which are selected based on their high similarity for two or three of the following conditions: neighbourhood information (spatial location), morphological distance d (optional), and the gene expression profile correlation (Fig. 1e). The technical variation addressed by stSME includes gene-level "dropout" (mis-detection of lowly-expressed genes due to capture efficiency) and spot-level sequencing depth (total reads per spot). stSME is based on the assumption that two spots that are nearby and/or closely resemble one another morphologically have more similar gene expression than spots that are more distant and/or morphologically distinct. This premise is well supported by previous studies. Indeed, morphological features such as cell size, nuclei size, granularity, local density, and cytoplasm in H&E- stained images are highly correlated with gene expression[1–4]. A positive correlation in gene expression level between cells/spots that are localised next to each other (close physical distance) has also been described[5–7]. We further validated this assumption by comparing morphological similarity (between high-level representations of image feature vectors) and gene expression similarity, and also the spatial coordinate (Euclidean) distance in ST data (Fig. S19). We found a higher correlation between the three distance matrices and between nearby spots, or spots within an anatomical region, than with spots that are further away and/or outside of these regions (Fig. S19d-h).

When morphological distance is included, stSME uses a neural network[8] to extract high-level features from tissue image pixel values. We used the ResNet50 convolutional neural network model, pre-trained on ImageNet, an approach that has been successfully applied in biomedical image analysis previously[3,9]. Although trained using images from a non-biomedical domain, the ImageNet database is very comprehensive (>10M images, over 20,000 categories), and the extracted features are thus also relevant to biological images (e.g. shapes, colours). The extracted feature vectors are used to estimate morphological similarities between neighbouring spots by cosine similarity (see Methods section, Fig. 1e, Fig.S19d, Fig. S20). As shown in Fig. S20d, the cosine similarity distance is high between two similar images, and low between images that are visually different. The extracted features from tissue images contain useful biological information, as demonstrated by the correlation with select handcraft features (Fig. S20b) and the improved representation of anatomical brain regions compared to raw R, G, B signals (Fig. S20c).

With the validated assumptions and distance calculation approaches for the three data types, we next select reference spots for each spot to correct for technical variation *and* to increase tissue coverage (through pseudo-spots). Our selection method represents an important improvement over simple neighbour smoothing approaches, which use spots immediately nearby. Specifically, stSME allows non-neighbouring spots that are highly similar in morphology and gene expression to also be used as the reference (Fig. 1e). We established three key criteria to find and apply more biologically relevant spots in stSME: *1)* location (those within a defined physical distance; (Fig. S19f), *2)* morphological similarity (Fig. S19d), and *3)* highly correlated gene expression profiles (likely to be spots of the same cell type; Fig. S19e). By default, three reference spots with the highest stSME correction weights are used (Fig. S19c), but stLearn stSME allows the user to increase the number of reference spots to suit more homogeneous tissues. We found that combining these three criteria increased the power to distinguish spots at different functional regions of a breast cancer tissue section (Fig. S19g, h).

stLearn's stSME approach can also enhance the normalisation process to account for technical variation between spots within a tissue. This result derives from two factors. First, as discussed above, stSME provides a validated means to recover zero values. Here, a 'zero-expression' value for a gene in one spot occurs only if all of its reference spots also have zero expression for the same gene (Fig. 1e). Assessing again the Visium breast cancer dataset, we found that stSME resulted in a significant reduction in the number of spots that contain high numbers of genes with zero values (Fig. 6c). Notably, most spots still have genes with zero values after stSME processing, indicating that stSME does not over-correct, as this would have led to most genes becoming 'non-zero' genes (Fig. 6c, bottom). stSME correction also does not change the overall distribution of total genes (Fig. 6c, top).

2 stSME improves unsupervised clustering, detection of rarer cell types and/or anatomical structures in the mouse brain

The benefits stSME's imputation strategy resulted in improved clustering results, enabling a better detection of cell types and/or anatomical regions. This became apparent when we evaluated SMEclust performance in resolving anatomical structures within Visium mouse brain ST data, using the well-characterised Allen Mouse Brain Atlas[10] for reference annotations. For example, when we performed Louvain clustering[11] on the stSME-normalised matrix (hereby referred to as SMEclust) for the mouse brain, and then compared results to four other clustering methods for either single-cell or spatial data[12–15], SMEclust was the only method that could distinguish between some of the smaller substructures within the hippocampus, specifically the Cornu Ammonis (CA) 3 (cluster 17) and CA1 (cluster 6) regions; SMEclust also reliably detected the dentate gyrus (DG, cluster 7), while Seurat[12], Giotto[15], BayesSpace[13] and SpaGCN[14] could not (Fig. S22a, c, d). These outcomes validate our stSME approach more generally, in that they demonstrate that it does not reduce but rather increases the sensitivity of detecting specific

cell types and/or regions at a higher resolution (Fig. 6b,d,g).

3 SMEclust accurately defines human brain cortex regions

In addition to the mouse brain, we also took advantage of existing ST data for human cortex to compare the performance of SMEclust against a set number of other spatial clustering methods, all up using n=12 datasets[16] and six clustering quality metrics (Fig. S22e,f; Fig. S23). We found that, across a comprehensive range of comparable parameter options, SMEclust outperformed several other clustering methods, including SpatialLIBD[16], SPATA[17], Seurat[18], and Giotto[15]; SMEclust performance was comparable to that of SpaGCN and BayesSpace but its variance was significantly smaller (two-sided F-test <0.05), suggesting SMEclust performance to be more stable. Performance metrics otherwise showed SMEclust to be more consistent with the "ground truth" histological annotation (Fig. S22f; Fig. S23). For localised, more homogeneous tissue regions (e.g. where neighbouring cells belong to the same cluster), smoothing methods perform generally well. However, for many cases where there are discrete layers and/or fine anatomical substructures, smoothing methods like BayesSpace[13] tend to produce over-smoothing effects, which is exemplified by it finding large groups of neighbouring cells that it classifies as not belonging to the cluster(s) representing the specific cortical layer (Fig. S22f; Fig. S23).

4 Finding cell states and gene markers in human breast cancer

To also assess stSME in a more applied clinical (pathology) setting, we similarly applied the SMEclust algorithm to the breast cancer dataset. Here, cell types and/or biological states are determined by disease stage (Fig. 6g; Fig. S24), which is different to the naïve mouse and human brain case studies presented above. All of the major invasive (IDC) and ductal carcinoma *in situ* (DCIS) clusters detected by a professional expert pathologist were also detected by SMEclust (Fig. 6g, Fig. S24). SMEclust did, however, also detect additional clusters that a trained pathologist could not. Many of these additional clusters were small yet molecularly distinct, with localised expression of both known and potentially new biomarkers for each cell type (Fig. S24, Fig. S19).

Lastly, it is also important to point here that stLearn's stSME use of spatial information to inform sub-clustering offers an innovative way to address the challenge of having to arbitrarily select the clustering resolution - a problem commonly encountered in single cell and spatial analysis. For example, in the human breast cancer dataset, only a single ductal cancer cluster was identified when using gene expression alone (Fig. S8b). However, the spatial separation of cells within this large cluster provided the supporting evidence to justify splitting this cluster into three sub-clusters (or clades) with transcriptionally different states that also followed different PSTS trajectories (Fig. S8d and Fig. S10, S11).

Overall, we conclude that SMEclust is able to accurately find anatomical (sub-)structures and/or cell states within a given tissue, across multiple settings and/or contexts, and to also reveal the specific gene markers that they express (Fig. S24).

References

1. Cutiongco, M. F. A., Jensen, B. S., Reynolds, P. M. & Gadegaard, N. Predicting gene expression using morphological cell responses to nanotopography. *Nat Commun* **11**, 1384 (2020).
2. Nassiri, I. & McCall, M. N. Systematic exploration of cell morphological phenotypes associated with a transcriptomic query. *Nucleic Acids Res* **46**, e116 (2018).
3. He, B. *et al.* Integrating spatial gene expression and breast tumour morphology via deep learning. *Nat Biomed Eng* **4**, 827–834 (2020).
4. Schmauch, B. *et al.* A deep learning model to predict RNA-Seq expression of tumours from whole slide images. *Nat Commun* **11**, 3877 (2020).
5. Svensson, V., Teichmann, S. A. & Stegle, O. SpatialDE: identification of spatially variable genes. *Nat. Methods* **15**, 343–346 (2018).
6. Armingol, E. *et al.* Inferring the spatial code of cell-cell interactions and communication across a whole animal body. *bioRxiv* DOI: 10.1101/2020.11.22.392217 (2020). https://www.biorxiv.org/content/early/2020/11/22/2020.11.22.392217.full.pdf.
7. Niethamer, T. K. *et al.* Defining the role of pulmonary endothelial cell heterogeneity in the response to acute lung injury. *Elife* **9** (2020).
8. He, K., Zhang, X., Ren, S. & Sun, J. Deep residual learning for image recognition. In *2016 IEEE Conference on Computer Vision and Pattern Recognition (CVPR)*, 770–778, DOI: 10.1109/CVPR.2016.90 (2016).
9. Shi, F. *et al.* Review of artificial intelligence techniques in imaging data acquisition, segmentation and diagnosis for covid-19. *IEEE reviews biomedical engineering* (2020).
10. Lein, E. S. *et al.* Genome-wide atlas of gene expression in the adult mouse brain. *Nature* **445**, 168–176 (2007).
11. Subelj, L. & Bajec, M. Unfolding communities in large complex networks: combining defensive and offensive label propagation for core extraction. *Phys Rev E Stat Nonlin Soft Matter Phys* **83**, 036103 (2011).
12. Stuart, T. *et al.* Comprehensive Integration of Single-Cell Data. *Cell* **177**, 1888–1902 (2019).
13. Zhao, E. *et al.* Spatial transcriptomics at subspot resolution with BayesSpace. *Nat Biotechnol* **39**, 1375–1384 (2021).
14. Hu, J. *et al.* SpaGCN: Integrating gene expression, spatial location and histology to identify spatial domains and spatially variable genes by graph convolutional network. *Nat Methods* **18**, 1342–1351 (2021).
15. Dries, R. *et al.* Giotto: a toolbox for integrative analysis and visualization of spatial expression data. *Genome Biol* **22**, 78 (2021).
16. Maynard, K. R. *et al.* Transcriptome-scale spatial gene expression in the human dorsolateral prefrontal cortex. *Nat Neurosci* **24**, 425–436 (2021).
17. Kueckelhaus, J. *et al.* Inferring spatially transient gene expression pattern from spatial transcriptomic studies. *bioRxiv* DOI: 10.1101/2020.10.20.346544 (2020). https://www.biorxiv.org/content/early/2020/10/21/2020.10.20.346544.full.pdf.
18. Stuart, T. *et al.* Comprehensive integration of single-cell data. *Cell* **177**, 1888–1902 (2019).
