# Supplementary material for: Robust mapping of spatiotemporal trajectories and cell–cell interactions in healthy and diseased tissues
Source: Nat Commun. 2023 Nov 25;14:7739. doi: 10.1038/s41467-023-43120-6 (PMC10676408; doi:10.1038/s41467-023-43120-6)
Supplement: Supplementary file 2 — Reporting Summary [file 41467_2023_43120_MOESM2_ESM.pdf]

Corresponding author(s): Quan Nguyen, Marc Ruitenberg

Last updated by author(s): 14/06/2023

Reporting Summary

Nature Portfolio wishes to improve the reproducibility of the work that we publish. This form provides structure for consistency and transparency in reporting. For further information on Nature Portfolio policies, see our Editorial Policies and the Editorial Policy Checklist.

Statistics

For all statistical analyses, confirm that the following items are present in the figure legend, table legend, main text, or Methods section.

n/a	Confirmed	
☐	☒	The exact sample size (*n*) for each experimental group/condition, given as a discrete number and unit of measurement
☐	☒	A statement on whether measurements were taken from distinct samples or whether the same sample was measured repeatedly
☐	☒	The statistical test(s) used AND whether they are one- or two-sided<br>*Only common tests should be described solely by name; describe more complex techniques in the Methods section.*
☐	☒	A description of all covariates tested
☐	☒	A description of any assumptions or corrections, such as tests of normality and adjustment for multiple comparisons
☐	☒	A full description of the statistical parameters including central tendency (e.g. means) or other basic estimates (e.g. regression coefficient) AND variation (e.g. standard deviation) or associated estimates of uncertainty (e.g. confidence intervals)
☐	☒	For null hypothesis testing, the test statistic (e.g. *F*, *t*, *r*) with confidence intervals, effect sizes, degrees of freedom and *P* value noted<br>*Give P values as exact values whenever suitable.*
☒	☐	For Bayesian analysis, information on the choice of priors and Markov chain Monte Carlo settings
☐	☒	For hierarchical and complex designs, identification of the appropriate level for tests and full reporting of outcomes
☐	☒	Estimates of effect sizes (e.g. Cohen's *d*, Pearson's *r*), indicating how they were calculated

Our web collection on statistics for biologists contains articles on many of the points above.

Software and code

Policy information about availability of computer code

Data collection	bcl2fastq V2.7.0 and SpaceRanger V1.0.0 was used for mapping spatial transcriptomics data of the human skin samples and mouse brain samples. Zeiss Zen Blue 3 for immunofluorescence imaging data.
Data analysis	stLearn software is implemented using Python and the source code is available at https://github.com/BiomedicalMachineLearning/stLearn (stLearn version 1.0.1 was used). Detailed tutorials can be found at https://stlearn.readthedocs.io/. The public Github repository of the interactive version of stLearn can be accessed at https://github.com/BiomedicalMachineLearning/stlearn_interactive. Code to reproduce figures presented in this paper is available at https://github.com/BiomedicalMachineLearning/stlearn_manuscript.<br><br>The following open-source software programs were implemented in the analysis and benchmarking workflow:<br>scanpy V.1.5.1<br>networkx V.2.4<br>anndata V.0.7.1<br>tensorflow V2.2.0<br>CellChat_1.1.3,<br>SingleCellSignalR_1.4.0,<br>squidpy v1.1.2,<br>CellPhoneDB v2.1.7,<br>NATMI (2021),<br>Variogram (SciKit-Gstat 1.0.0)<br>slingshot V1.9.1<br>monocle3 V1.0.0

CytoTRACE V1.0.0; DBSCAN (scikit-learn 1.0.2); SpaGCN: 1.2.0; BayesSpace: 1.1.4
Seurat: 4.0.5
SPATA: 0.1.0
Giotto: 1.1.0
bcl2fastq:2.7.0
ForceAtlas2
gseapy1.0.5
Fiji2.1.0
StereoInvestigator

For manuscripts utilizing custom algorithms or software that are central to the research but not yet described in published literature, software must be made available to editors and reviewers. We strongly encourage code deposition in a community repository (e.g. GitHub). See the Nature Portfolio guidelines for submitting code & software for further information.

Data

Policy information about availability of data

All manuscripts must include a data availability statement. This statement should provide the following information, where applicable:

- Accession codes, unique identifiers, or web links for publicly available datasets
- A description of any restrictions on data availability
- For clinical datasets or third party data, please ensure that the statement adheres to our policy

Sequencing data (and associated H\&E images), both raw and processed, for experimental datasets generated as part of this study, have been deposited to the NCBI's Gene Expression Omnibus (GEO) database under the accession code GSE236171 (https://www.ncbi.nlm.nih.gov/geo/query/acc.cgi?acc=GSE236171) and made publicly available. All other imaging data from RNAscope and immunofluorescence-based histological studies can be made available upon request.

Our simulated dataset can be reproducibly generated using code available in the stLearn manuscript GitHub page at https://github.com/BiomedicalMachineLearning/stlearn_manuscript/tree/main/Main_figure_6_CCI_with_Sup/scripts/X6_breast_cancer_simulation

We also used publicly available datasets, as described below (and in the manuscript).

Visium human brain dataset:We downloaded the count matrix, annotation and spatial data from Maynard et al https://github.com/LieberInstitute/HumanPilot]. There are 47,681 spots (12 samples), with an average of 3462 UMIs detected per spot and an equivalent of 1734 genes per spot.

Visium coronal mouse brain dataset:We downloaded the count matrix, annotation and spatial data from 10x Genomics public dataset at https://support.10xgenomics.com/spatial-gene-expression/datasets/1.1.0/. In total, there are 2,702 spots, with a median of 28,944 UMIs andand 6,018 genes detected per spot.

Visium human breast cancer dataset:We obtained the Human Breast Cancer Visium dataset from the 10X Genomics website (https://support.10xgenomics.com/spatial-gene-expression/datasets/1.0.0/V1_Breast_Cancer_Block_A_Section_1. It contains 3,813 spots under tissuewith median UMI counts per spot of 17,531 and median genes per spot of 5,394.

Sci-Space mouse embryo brain dataset:We downloaded the count matrix, spatial data and metadata of the sci-Space mouse embryo brain dataset45from National Center for Biotechnology Information (NCBI) under the accession number GSE166692. From the raw data of 121,365 cells(average of 2,514 UMIs and 1,231 genes per cell), we subsetted to keep all of 15,466 cells in categories "Neuron", "Glial Cells",and "Radial glia" (keeping cells with fewer than 7,000 genes and at least 200 genes).

seqFISH+ mouse embryo sub-ventricular zone (SVZ) dataset: seqFISH+ data of mouse brain (2963 cells) with an average of 3338 genes per cell were downloaded from website(https://github.com/CaiGroup/seqFISH-PLUS/blob/master/sourcedata.zip), (accessed February 2022).

Slide-seq mouse olfactory bulb:Slide-seq data15containing 47,573 cells and 20,572 genes were downloaded from website (https://www.dropbox.com/s/cs6pii5my4p3ke3/mouse_hippocampus_reference.rds?dl=0), (accessed February 2022).

Human research participants

Policy information about studies involving human research participants and Sex and Gender in Research.

Reporting on sex and gender

Use the terms sex (biological attribute) and gender (shaped by social and cultural circumstances) carefully in order to avoid confusing both terms. Indicate if findings apply to only one sex or gender; describe whether sex and gender were considered in study design whether sex and/or gender was determined based on self-reporting or assigned and methods used. Provide in the source data disaggregated sex and gender data where this information has been collected, and consent has been obtained for sharing of individual-level data; provide overall numbers in this Reporting Summary. Please state if this information has not been collected. Report sex- and gender-based analyses where performed, justify reasons for lack of sex- and gender-based analysis.

Population characteristics

Describe the covariate-relevant population characteristics of the human research participants (e.g. age, genotypic information, past and current diagnosis and treatment categories). If you filled out the behavioural & social sciences study design questions and have nothing to add here, write "See above."

Recruitment	*A skin biopsy sample from one patient diagnosed with basal cell carcinoma (BCC) collected at the Dermatology Department of the Princess Alexandra Hospital in 2019. No bias was aware that may be to impact results.*
Ethics oversight	The study was approved by The University of Queensland Human Research Ethics Committee (HREC-ll-QPAH-477, The University of Queensland, Clearance No. 2012000052). No compensation was required and provided.

Note that full information on the approval of the study protocol must also be provided in the manuscript.

Field-specific reporting

Please select the one below that is the best fit for your research. If you are not sure, read the appropriate sections before making your selection.

☒ Life sciences ☐ Behavioural & social sciences ☐ Ecological, evolutionary & environmental sciences

For a reference copy of the document with all sections, see nature.com/documents/nr-reporting-summary-flat.pdf

Life sciences study design

All studies must disclose on these points even when the disclosure is negative.

Sample size	We applied a strategy to gain statistical robustness by using orthogonal validation from applying multiple orthogonal methods (both analytical and experimental) for the same set of samples, from a diverse range of datasets representing different biological systems, and from simulation. For validation of our results, we used multiple independent public datasets. We used 2x breast cancer sections (total 3,813 spots), 12x human brain samples (Maynard et al, 2020; 2x technical replicates as adjacent tissues, 2x spatial replicates from 3x patients; total 47,680 spots), 4x traumatic brain injury samples (6,337 sots), 2x sci-Space datasets for embryonic E14 samples (total 121,365 cells; used 15,466 cells), 2x human skin cancer samples (1,179 spots), 2x seqFISH+ datasets for mouse sub-ventricular zone tissues (2,963 cells), 2x Slide-seq datasets for mouse olfactory bulb (47,573 cells) . Imaging experimental validation of the PSTS algorithm contained 4 replicates per time point, and a total of 6 tine points.<br><br>Our analyses focused on finding spatial patterns within a tissue, which gained statistical power from the large number of spots/cells. From one transcriptomics tissue section, hundreds to thousands of spatial spots are measured. The number of spots in each cell type can be considered as the number of technical replicates of the same cell type within a tissue. Using these replicates, we can account for technical variation, such as sequencing depth and PCR-amplification bias when comparing clusters within the same tissue section. Using simulation data of single cell and spatial data, we calculated that to find a rare population with a frequency of 1% of a sample of spots with 95% detection power we needed 628 spots. All datasets we used had more than >1000 spots, as described above.<br><br>We also simulated spatial data using a probabilistic approach for breast cancer tissues. We applied this approach for assessing cell cell interaction analysis and for comparing with other methods.
Data exclusions	For spatial transcriptomics data, we applied a quality control step for excluding lowly expressed genes (genes detected in fewer than 3 spots) and spots with low transcriptome diversity (fewer than 100 genes). For the analysis of Sci-Space data, we used a data subset that includes cell types involved in neuronal differentiation and migration.
Replication	For clustering analysis, we tested three different mouse brain sections and used well characterised anatomical regions as the ground truth. We used 12 human brain datasets and compared results to independent pathologist's annotation reference. For trajectory analysis, we used three different biological systems (mouse brain injury, human breast cancer, and mouse development cortex). For cell to cell interaction analysis, we tested human breast cancer, human skin cancer, mouse cortex sub-ventricular zone, and mouse hippocampus. In each case above, we have at least two samples as biological replicates, and we had more than 1000 cells/spots for each condition. Further, we developed a simulation strategy for assessing assumptions and performance. For further assessment, we compared our algorithms with five other independent methods, across a range of assessment criteria. All replications were successfully used in the analysis.
Randomization	We thoroughly assessed false discovery as well as the robustness of null distribution (for example in the case of cell cell interaction analysis, we performed over one million permutation test to randomize genes and cell types). Our randomization strategy involves random shuffling and pairing of genes, cells, and spatial locations.
Blinding	All analyses done in this study were data-driven and unsupervised [illegible] For example, we applied unsupervised clustering to identify cell types, with no prior information about the expected number of cell types present in the dataset.

Reporting for specific materials, systems and methods

We require information from authors about some types of materials, experimental systems and methods used in many studies. Here, indicate whether each material, system or method listed is relevant to your study. If you are not sure if a list item applies to your research, read the appropriate section before selecting a response.

Materials & experimental systems

n/a	Involved in the study
☒	☐ Antibodies
☒	☐ Eukaryotic cell lines
☒	☐ Palaeontology and archaeology
☐	☒ Animals and other organisms
☒	☐ Clinical data
☒	☐ Dual use research of concern

Methods

n/a	Involved in the study
☒	☐ ChIP-seq
☒	☐ Flow cytometry
☒	☐ MRI-based neuroimaging

Animals and other research organisms

Policy information about studies involving animals; ARRIVE guidelines recommended for reporting animal research, and Sex and Gender in Research

Laboratory animals	Three-month-old female C57BL/6J mice were used. On a 12-hour light-dark cycle in individually ventilated cages.
Wild animals	The study did not involve wild animal.
Reporting on sex	Female mice were used
Field-collected samples	The study did not involve samples collected from the field.
Ethics oversight	All experiments were conducted in accordance with the Australian Code for the Care and Use of Animals for Scientific Purposes, with approval from The University of Queensland Animal Ethics Committee.

Note that full information on the approval of the study protocol must also be provided in the manuscript.
